# Cardiac *Slc25a49*‐Mediated Energy Reprogramming Governs Doxorubicin‐Induced Cardiomyopathy through the G6P–AP‐1–Sln Axis

**DOI:** 10.1002/advs.202502163

**Published:** 2025-04-04

**Authors:** Sitong Wan, Jingyi Qi, Yi Xia, Chang Fan, Teng Xu, Xu Zhang, Jiaxin Shi, Chenxuan Wang, Yitong Cheng, Dongyuan Zhang, Rong Liu, Yinhua Zhu, Changchang Cao, Dekui Jin, Peng An, Yongting Luo, Junjie Luo

**Affiliations:** ^1^ Key Laboratory of Precision Nutrition and Food Quality Department of Nutrition and Health China Agricultural University Beijing 100193 China; ^2^ Food Science Institute Zhejiang Academy of Agricultural Sciences Hangzhou 310021 China; ^3^ State Key Laboratory of Cardiovascular Disease Fuwai Hospital National Center for Cardiovascular Diseases Chinese Academy of Medical Sciences and Peking Union Medical College Beijing 100037 China; ^4^ Department of General Practice The Third Medical Center of Chinese PLA General Hospital Beijing 100039 China

**Keywords:** cardiomyopathy, doxorubicin, energy reprogramming, mitochondria, Slc25a49–G6P–AP‐1–Sln axis

## Abstract

Doxorubicin (Dox), a potent antitumor drug, is linked to cardiac toxicity. Few mechanism‐based therapies against cardiotoxicity are available. Dysfunction in mitochondrial energy metabolism contributes to Dox‐induced cardiomyopathy. It is aimed at exploring the association between specific mechanism of energy reprogramming and Dox‐induced cardiomyopathy. Cardiac‐specific ablation of *Slc25a49* mice are generated by crossing *Slc25a49^flox/flox^* mice with *Myh6‐Cre* mice. *Slc25a49^HKO^* mice or *SLC25A49^KD^* cardiomyocytes is treated with Dox. Echocardiography, histological analysis, transmission electron microscopy, bulk RNA sequencing, cell bioenergetic profiling, metabolomics test, chromatin immunoprecipitation, and dual‐luciferase reporter assay are conducted to delineate the phenotype and elucidate the molecular mechanisms. Specific ablation of *Slc25a49* in cardiomyocytes leads to exacerbated Dox‐induced cardiomyopathy, characterized by compromised mitochondrial respiration enhanced glycolysis and increased glycolytic metabolite glucose‐6‐phosphate (G6P) levels, subsequently activating the activator protein‐1 (AP‐1) complex. The stimulation of the G6P–AP‐1 axis intensifies myocardial damage via transcriptionally regulating Sarcolipin (Sln) expression. Strikingly, targeting of this axis with the AP‐1 inhibitor T‐5224 effectively improves survival and enhances cardiac function in Dox‐induced cardiomyopathy. This study provides mechanistic insights into energy reprogramming that permits myocardial dysfunction, and thus provides a proof of concept for antienergy reprogramming therapy for Dox‐induced cardiomyopathy through directly modulating G6P–AP‐1–Sln axis.

## Graphical abstract

Doxorubicin‐induced cardiomyopathy involves mitochondrial energy metabolism dysfunction, exacerbated by cardiomyocyte‐specific *Slc25a49* deficiency via oxidative phosphorylation (OXPHOS) suppression and glycolysis activation. The Slc25a49–glucose‐6‐phosphate (G6P)–activator protein‐1 (AP‐1) axis drives myocardial injury by upregulating Sln, disrupting calcium homeostasis. AP‐1 inhibitor T‐5224 rescues survival, cardiac function, and morphology—revealing an energy reprogramming mechanism with therapeutic promise.

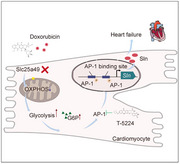

## 1 Introduction

Doxorubicin (Dox), a potent antineoplastic agent with a favorable therapeutic index, holds a distinctive role in cancer therapy. It is utilized in the treatment of multiple cancers, including but not limited to breast cancer, lymphoma, and leukemia.^[^ ^1^ ^]^ Nonetheless, the clinical utility of Dox is constrained by its cardiotoxicity, which encompasses initial anomalies in myocardial damage markers and the gradual onset of cardiomyopathy, heart failure, and arrhythmias.^[^ ^2^ ^]^ The etiology of Dox‐induced heart failure is a multifaceted process, with accumulating data indicating a significant correlation with mitochondrial dysfunction and disrupted energy metabolism.^[^ ^3^ ^]^ Mitochondria are central to the energetics of cardiomyocytes. In the typical process of oxidative phosphorylation (OXPHOS), mitochondria generate substantial quantities of adenosine triphosphate (ATP), which powers the contraction and relaxation of cardiomyocytes.^[^ ^4^ ^]^

The disturbance of mitochondrial energy metabolism and upregulation of glycolysis are the primary culprits in Dox‐induced cardiotoxicity.^[^ ^5^ ^]^ In typical cardiomyocytes, mitochondrial energy metabolism predominates, enabling the effective production of substantial quantities of ATP via OXPHOS.^[^ ^6^ ^]^ Dox treatment drastically interferes with mitochondrial energy metabolism and induces cardiotoxicity.^[^ ^7^ ^]^ Specifically, Dox disrupts the expression and function of electron transport chain (ETC)‐related genes, including the action of complexes I–IV, hence obstructing the formation of normal proton electrochemical gradients.^[^ ^8^ ^]^ This subsequently hinders OXPHOS, leading to a substantial reduction in ATP production efficiency. Simultaneously, a considerable quantity of free radicals generated by Dox instigating mitochondrial lipid peroxidation, compromising membrane integrity and permeability, ultimately leading to mitochondrial enlargement, rupture, and altered energy metabolism.^[^ ^9^ ^]^ In addition, impaired mitochondrial OXPHOS is coupled with enhanced glycolysis in Dox‐induced cardiotoxicity,^[^ ^10^ ^]^ serving as a compensatory strategy to mitigate the energy deficit resulting from defective mitochondrial OXPHOS.^[^ ^11^ ^]^ Collectively, the energy reprogramming of decreased mitochondrial energy metabolism and compensatory increase in glycolysis levels is typically observed in Dox‐induced cardiotoxicity. Nonetheless, the relationship and precise mechanisms between mitochondrial energy metabolism and Dox‐induced cardiomyopathy have yet to be completely clarified.

Mitochondrial transmembrane proteins are essential for the normal functioning of mitochondria and may regulate OXPHOS, oxidative stress, and reactive oxygen sepsis (ROS).^[^ ^12^ ^]^ Proteins expressed by the mitochondrial carrier family, known as the SLC25 gene family, are chiefly responsible for the transport of metabolites across the mitochondrial membrane, which is essential for sustaining mitochondrial function and cellular energy metabolism.^[^ ^13^ ^]^ For instance, mutations in the *Slc25a46* gene correlate with abnormal mitochondrial shape and compromised mitochondrial function in the fibroblasts generated from patients, subsequently impacting the oxidative stress and ATP synthesis.^[^ ^14^ ^]^ Slc25a51 has been shown to function as an importer of the energy substrate NAD^+^.^[^ ^15^ ^]^ Loss of *Slc25a51* gene in cardiomyocytes induces cardiac hypertrophy by impairing complex I respiration and mitochondrial NAD^+^ level.^[^ ^16^ ^]^ Slc25a49 (Mtch1) is a penta‐spanning transmembrane protein situated in the outer mitochondrial membrane.^[^ ^17^ ^]^ Our recent study revealed that *SLC25A49* deficiency disrupted OXPHOS and energy production, signifying its crucial function in cellular energy metabolism,^[^ ^18^ ^]^ with a metabolic abnormal phenotype similar to Dox‐induced cardiac mitochondrial damage. Thus, an energy reprogramming mouse model may be constructed based on *Slc25a49* deficiency to explore the mechanism by which energy reprogramming regulates Dox‐induced cardiomyopathy.

This study aims to elucidate the regulatory mechanisms behind Dox‐induced cardiomyopathy and identify novel therapeutic targets for clinical intervention. We demonstrate that a Slc25a49–glucose‐6‐phosphate (G6P)–activator protein‐1 (AP‐1)–Sln axis in cardiomyocytes triggers Dox‐induced cardiac remodeling through energetic reprogramming. Disruption of this axis blunts cardiac remodeling and produces a marked attenuation of cardiac injury. These findings uncover previously uncharacterized energetic reprogramming during cardiac remodeling, and suggest that blocking of this axis represents a potential avenue for developing effective therapies against Dox‐induced cardiotoxicity.

## 2 Results

### 2.1 Cardiac‐Specific *Slc25a49* Ablation Exacerbates Dox‐Induced Cardiomyopathy

**Figure 1:**
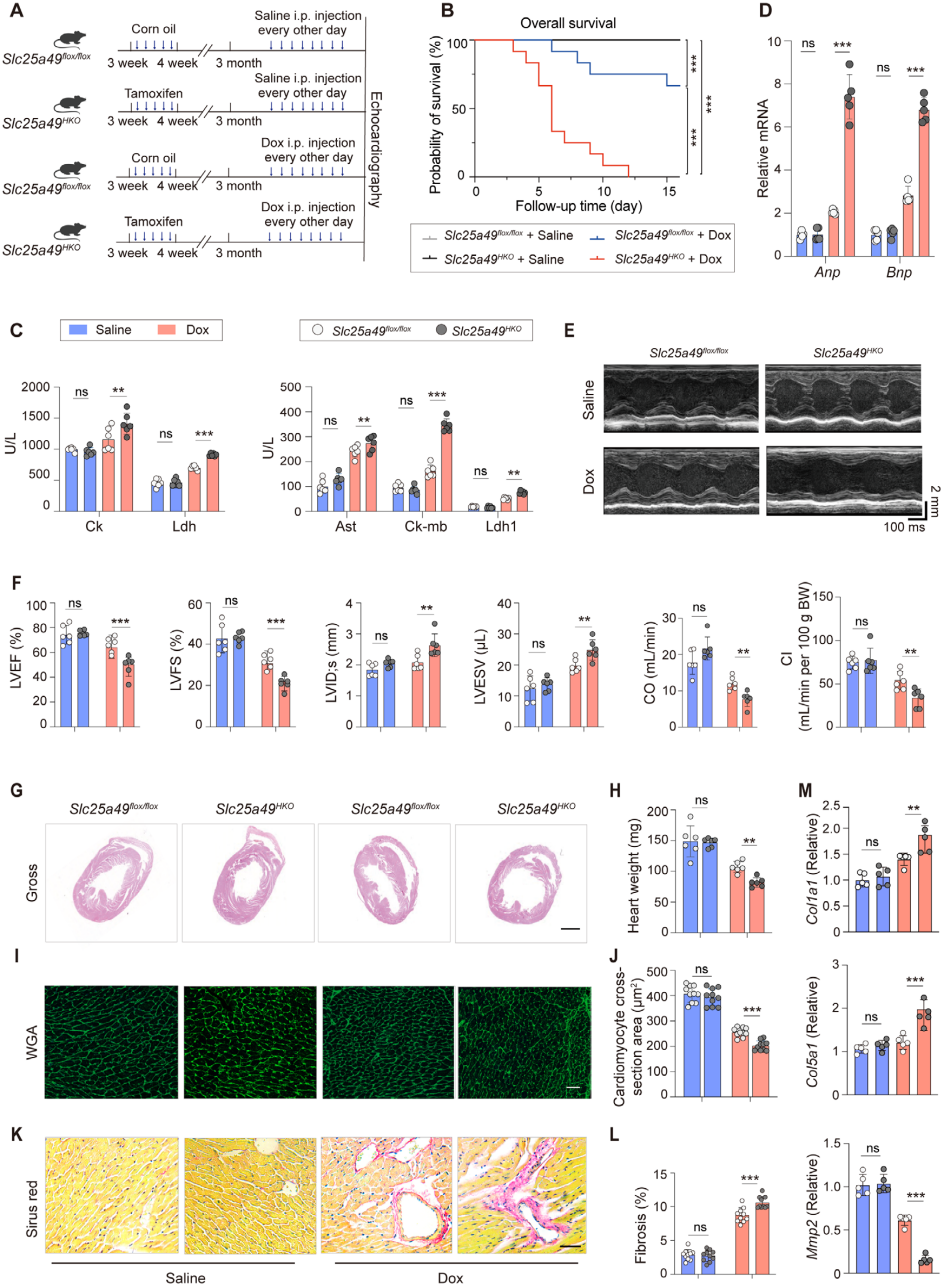
*Slc25a49^HKO^* exacerbates Dox‐induced cardiomyopathy. A) Experiment timeline in vivo. B) Kaplan–Meier plot showing increased mortality in Dox‐treated *Slc25a49^HKO^* mice (*n* = 12 for each group). C) Serum levels of Ck, Ldh, Ast, Ck‐mb, and Ldh‐1 were measured in *Slc25a49^flox/flox^* and *Slc25a49^HKO^* mice treated with Corn oil or Dox at 3 months (*n* = 6 for each group). D) Real‐time quantitative PCR analysis of *Anp* and *Bnp* mRNA expression in four groups of mice at 3 months (*n* = 5 for each group). E) Echocardiography of four groups of mice at 3 months. F) Echocardiography parameters of (E) were calculated (*n* = 6 for each group). G) Horizontal morphology of *Slc25a49^flox/flox^* and *Slc25a49^HKO^* hearts with or without Dox‐treatment (scale bar = 1 mm). H) Comparison of heart weight among four groups of mice (*n* = 6 for each group). I) Wheat germ agglutinin (WGA) staining to compare sectional cardiomyocyte size in four groups of mice (Scale bar = 100 µm). J) Quantification of cardiomyocyte area in (I) using Image J (*n* = 10 for each group). K) Representative Sirus Red staining among four groups of mice (Scale bar = 50 µm). L) Quantification of K) (*n* = 10 for each group). M) Real‐time quantitative PCR analysis of *Col1a1*, *Col3a1*, and *Mmp2* mRNA expression in four groups of mice at 3 months (*n* = 5 for each group). Ck, creatine kinase; Ldh, lactic dehydrogenase; Ast, aspartate aminotransferase; Anp, atrial natriuretic peptide; Bnp, brain natriuretic peptide; LVEF, left ventricular ejection fraction; LVFS, left ventricular fraction shortening; LVID;s, left ventricular internal dimension at systole; LVESV, left ventricular end‐systolic volume; CO, cardiac output; and CI, cardiac input. Data were presented as means ± SD, and analyzed by one‐way ANOVA test. ns, not significant, **P < 0.01, and ***P < 0.001.

To investigate the role of *Slc25a49* in Dox‐induced cardiomyopathy, we generated mice with tamoxifen‐induced, cardiac‐specific *Slc25a49* gene knockout by interbreeding *Slc25a49^flox/flox^* mice with *Myh6‐Cre/ERT2* transgenic mice (*Slc25a49^flox/flox^*; *Myh6‐Cre/ERT2*; Figure S1A–C, Supporting Information). To obtain *Slc25a49^HKO^* or *Slc25a49^flox/flox^* (*Slc25a49^Con^*) mice, tamoxifen or corn oil was administered to *Slc25a49^flox/flox^*; *Myh6‐Cre/ERT2* mice via intraperitoneal (IP) injection at age 3 weeks for 5 consecutive days (**Figure** **1**A).

Male *Slc25a49^HKO^* and *Slc25a49^flox/flox^* mice were randomized to Dox (3 mg kg^−1^ IP every other day, accumulated 24 mg kg^−1^) or saline‐treated groups at 3 months to achieve chronic cardiomyopathy. *Slc25a49^HKO^* mice demonstrated markedly increased mortality compared to *Slc25a49^flox/flox^* mice, with all *Slc25a49^HKO^* mice succumbing by day 12 post‐Dox treatment (Figure 1B). Each group of mice underwent echocardiographic analysis and were euthanized on day 7 after Dox or saline‐treated for phenotype analysis. *Slc25a49* mRNA expression in Dox‐treated *Slc25a49^flox/flox^* mice heart has a significant upregulation than in *Slc25a49^flox/flox^* mice heart (Figure S1D, Supporting Information). By detecting the levels of myocardial enzyme markers in the serum, we found lactate dehydrogenase (Ldh), lactate dehydrogenase isoenzyme 1 (Ldh‐1), aspartate aminotransferase (Ast), creatine kinase (Ck), and creatine kinase isoenzyme (Ck‐mb) were all significantly further increased in *Slc25a49^HKO^* mice compared to *Slc25a49^flox/flox^* mice following Dox induction (Figure 1C). The deletion of cardiac‐specific *Slc25a49*, in conjunction with Dox treatment, also elevated the mRNA expression of embryonic cardiac genes (*Anp* and *Bnp*, Figure 1D), suggesting pathological cardiac remodeling. Serial echocardiographic analysis revealed that Dox treatment in *Slc25a49^flox/flox^* mice at 3 months decreased left ventricular ejection fractions (LVEF), left ventricular fractional shortening (LVFS), cardiac output (CO), and cardiac input (CI), with these metrics seeing more significant reduction in Dox‐treated *Slc25a49^HKO^* mice. Dox‐treated *Slc25a49^HKO^* mice showed a further significantly increase in left ventricular internal dimension at systole (LVIDs) and left ventricular end‐systolic volume (LVESV), but a decrease in heart weight, compared to *Slc25a49^flox/flox^* mice (Figure 1E–H). In the *Slc25a49^flox/flox^* heart, Dox treatment significantly decreased cardiomyocyte size and augmented perivascular and interstitial fibrosis, with these alterations exacerbated in the *Slc25a49^HKO^* heart (Figure 1I–L). Finally, we found that Dox‐treated cardiac‐specific *Slc25a49* deletion upregulated the *Col1a1* and *Col5a1* mRNA expression, and reduced *Mmp2* mRNA expression (Figure 1M). Thus, these findings suggest that cardiac *Slc25a49* deficiency exacerbates Dox‐induced cardiomyopathy.

### 2.2 *Slc25a49* Deletion Aggravates Dox‐Induced Cardiomyopathy Through Energy Reprogramming

**Figure 2:**
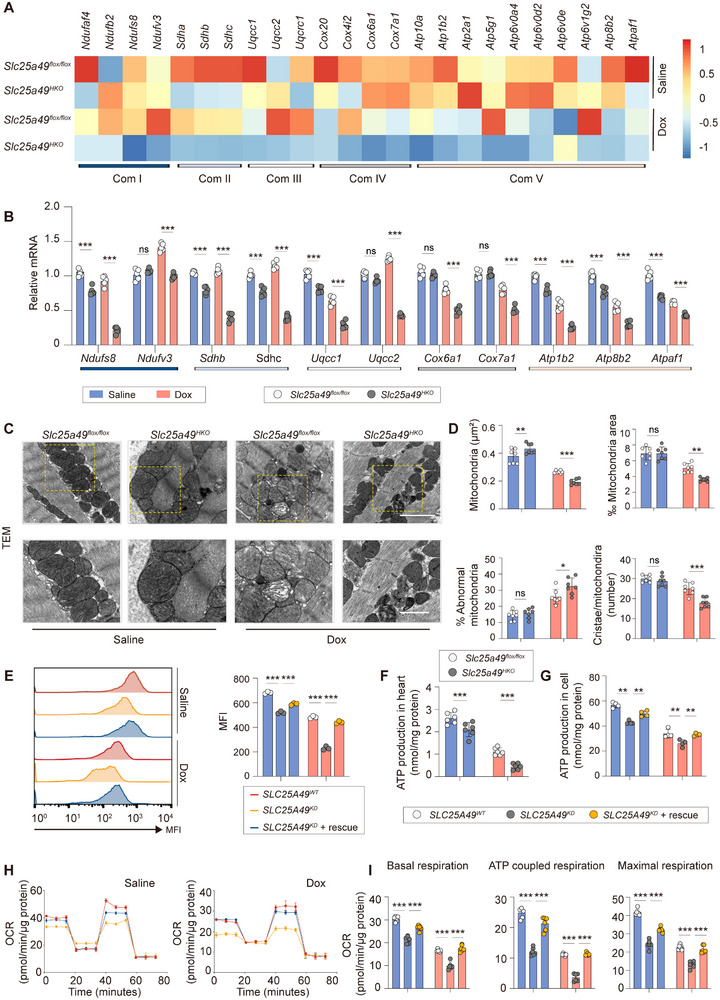
*Slc25a49^HKO^* inhibits OXPHOS with Dox‐treatment. A) Heatmap presenting differentially expressed mitochondrial respiratory ETC (Complex I‐V) genes in *Slc25a49^flox/flox^* and *Slc25a49^HKO^* hearts with or without Dox‐treatment at 3 months of age (*n* = 3 for each group). B) Real‐time quantitative PCR analysis for determining the mRNA expression of selected ETC complex genes in four groups of mice (*n* = 5 for each group). C) Representative transmission electron microscopy images of four groups of mice at 3 months (Top: 7392 scale bar = 1 µm; bottom: scale bar = 2 µm). D) Quantification of mitochondria‐related parameters from (C). Mitochondria/µm^2^ refers to the average number of mitochondria (*n* = 7 for each group); ‰ mitochondrial area refers to the ratio of mitochondrial area to image area (*n* = 7 for each group); % abnormal mitochondria refers to the percentage of abnormal mitochondria in individual samples (*n* = 7 for each group); cristae/mitochondira refers to the surface area of the inner mitochondrial membrane (*n* = 7 for each group). E) Measurement of mitochondrial membrane potential in AC16 clones of *SLC25A49^WT^*, *SLC25A49^KD^*, and *SLC25A49^KD^* + rescue treated with or without Dox for 24 h (Dox, 500 nm; *n* = 3 for each group). F) ATP content in four groups of mice at 3 months (*n* = 6 for each group). G) ATP content in six groups of AC16 clones *SLC25A49^WT^*, *SLC25A49^KD^*, *SLC25A49^KD^* + rescue treated with or without Dox for 24 h (Dox, 500 nm; *n* = 4 for each group). H) Real‐time monitoring the OCR in AC16 clones of *SLC25A49^WT^*, *SLC25A49^KD^*, and *SLC25A49^KD^* + rescue treated with or without Dox for 24 h (Dox, 500 nm; *n* = 6 for each group). I) OCR was measured in AC16 clones of *SLC25A49^WT^*, *SLC25A49^KD^*, and *SLC25A49^KD^* + rescue treated with or without Dox for 24 h (Dox, 500 nm; *n* = 6 for each group). OXPHOS, oxidative phosphorylation; ETC, electron transport chain; ATP, adenosine triphosphate; OCR, oxygen consumption rates. Data were presented as means ± SD, and analyzed by one‐way ANOVA test. ns, not significant, *P < 0.05, **P < 0.01, and ***P < 0.001.

**Figure 3:**
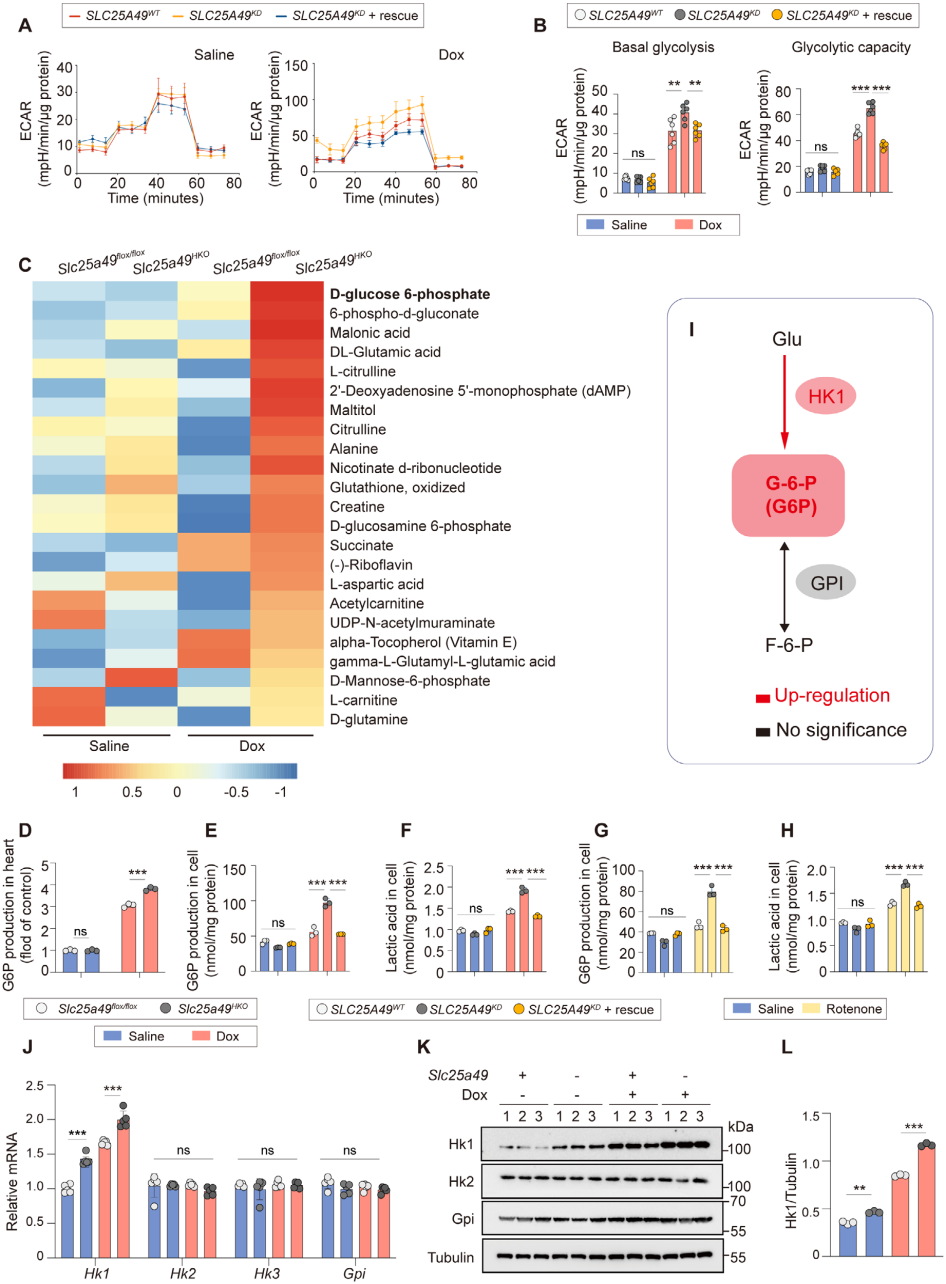
*Slc25a49 ^HKO^* upregulates glycolysis and accumulates G6P. A) Real‐time monitoring the ECAR in AC16 clones of *SLC25A49^WT^*, *SLC25A49^KD^*, and *SLC25A49^KD^* + rescue treated with or without Dox for 24 h (Dox, 500 nm; *n* = 6 for each group). B) ECAR was measured in AC16 clones of *SLC25A49^WT^*, *SLC25A49^KD^*, and *SLC25A49^KD^* + rescue treated with or without Dox for 24 h (Dox, 500 nm; *n* = 6 for each group). C) Heatmap showing differential metabolites in *Slc25a49^flox/flox^* and *Slc25a49^HKO^* hearts with or without Dox‐treatment at 3 months of age (*n* = 3 for each group). D) G6P levels in four groups of mice (*n* = 3 for each group). E) G6P levels in *SLC25A49^WT^*, *SLC25A49^KD^*, and *SLC25A49^KD^* + rescue hearts treated with or without Dox for 24 h (Dox, 500 nm; *n* = 3 for each group). F) Lactic acid levels in AC16 clones of *SLC25A49^WT^*, *SLC25A49^KD^*, and *SLC25A49^KD^* + rescue treated with or without Dox for 24 h (Dox, 500 nm; *n* = 3 for each group). G) G6P levels in AC16 clones of *SLC25A49^WT^*, *SLC25A49^KD^*, and *SLC25A49^KD^* + rescue treated with or without rotenone for 24 h (Rotenone, 1 µm; *n* = 3 for each group). H) Lactic acid levels in AC16 clones of *SLC25A49^WT^*, *SLC25A49^KD^*, and *SLC25A49^KD^* + rescue treated with or without rotenone for 24 h (Rotenone, 1 µm; *n* = 3 for each group). I) Schematic representation of G6P‐related changes in glycolysis. J). Real‐time quantitative PCR determining the mRNA expression of G6P‐related genes during glycolysis in four groups of mice (*n* = 5 for each group). K) Representative immunoblotting images showing G6P‐related protein during glycolysis in four groups of mice (*n* = 3 for each group). L) Quantification of (K). ECAR, extracellular acidification rate; HK1, hexokinase 1; G6P, glucose‐6‐phosphate; GPI, glucose‐6‐phosphate isomerase; F‐6‐P, fructose‐6‐phosphate. Data were presented as means ± SD, and analyzed by one‐way ANOVA test. ns, not significant, **P < 0.01, and ***P < 0.001.

To explore the mechanisms by which *Slc25a49* deletion aggravates Dox‐induced cardiomyopathy, we performed comprehensive RNA‐seq analysis on *Slc25a49^HKO^* and *Slc25a49^flox/flox^* hearts treated with saline or Dox. A broad downregulation of mitochondrial respiratory ETC genes was observed in Dox‐treated *Slc25a49^HKO^* hearts (**Figure** **2**A). As expected, the expression of selected ETC complex genes was further validated following Dox administration (Figure 2B and Figure S2A, Supporting Information). Besides, a reduction in mRNA expression of mitochondrial‐encoded OXPHOS‐related genes was noted in Dox‐treated *SLC25A49* knockdown (*SLC25A49^KD^*) cardiomyocytes (Figure S2B,C, Supporting Information), possibly due to mitochondrial abnormalities.^[^ ^20^ ^]^ Consequently, we evaluated mitochondrial ultrastructure via transmission electron microscopy and found that the deletion of *Slc25a49*, in conjunction with Dox treatment, led to aberrant mitochondrial shape and density relative to *Slc25a49^flox/flox^* mice (Figure 2C,D). The drop in mitochondrial membrane potential and ATP concentration in Dox‐treated *Slc25a49^HKO^* hearts or *SLC25A49^KD^* cardiomyocytes likewise indicated OXPHOS impairment (Figure 2E–G). Moreover, we utilized extracellular flux analysis to real‐time monitor mitochondrial respiratory capacity in vitro. Dox‐treated *SLC25A49^KD^* dramatically decreased basal, ATP production‐coupled, and maximal respiration, along with spare respiratory capacity (Figure 2H,I), while enhancing glycolysis and glycolytic capacity (**Figure** **3**A,B).

Metabolomics results revealed increased concentrations of glycolysis‐related metabolites, notably glucose‐6‐phosphate (G6P), in Dox‐treated *Slc25a49^HKO^* hearts (Figure 3C). We corroborated this conclusion by evaluating G6P levels both in vivo and in vitro. The levels of G6P consistently increased significantly in Dox‐treated *Slc25a49^HKO^* hearts or *SLC25A49^KD^* cardiomyocytes (Figure 3D–E). Additionally, elevated lactic acid levels signified the activation of glycolysis following *SLC25A49* knockdown (Figure 3F). A notable increase in G6P and lactate levels was observed in cardiomyocytes postrotenone treatment, suggesting that the burst of glycolysis may serve as an energy‐compensatory mechanism in response to compromised mitochondrial OXPHOS (Figure 3G,H). G6P is an endogenous intermediate of the glycolytic pathway, generated by hexokinase (HK) and eliminated by glucose‐6‐phosphate isomerase (GPI). Here, our results indicated that elevated HK1 levels, alongside stable GPI levels, led to a significant increase in G6P levels in Dox‐treated *Slc25a49^HKO^* hearts and *SLC25A49^KD^* cardiomyocytes (Figure 3I–L and Figure S3A–C, Supporting Information). These data suggest that *Slc25a49* deletion results in energy reprogramming by impaired mitochondrial OXPHOS and compensatory elevation of glycolysis, especially G6P levels, thereby exacerbating the progression of Dox‐induced cardiomyopathy.

### 2.3 G6P Enhances the Phosphorylation and Nuclear Translocation of AP‐1

**Figure 4:**
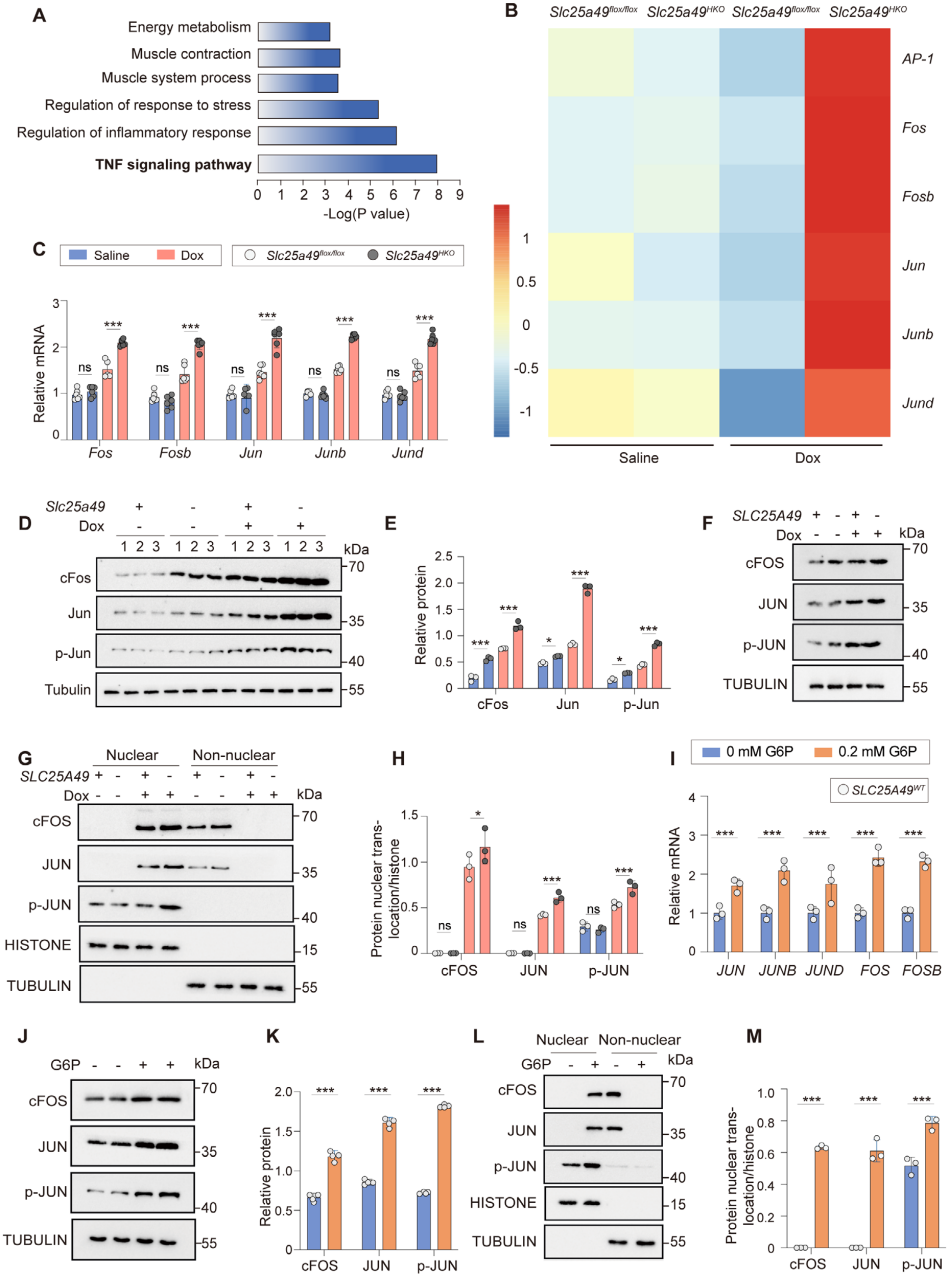
G6P enhances AP‐1 phosphorylation and nuclear translocation. A) KEGG enrichment analysis of RNA‐sequencing data from 3‐month‐old hearts of Dox‐treated *Slc25a49^flox/flox^* and *Slc25a49^HKO^* mice. B) Heatmap presenting differentially expressed AP‐1 family genes in *Slc25a49^flox/flox^* and *Slc25a49^HKO^* hearts with or without Dox‐treatment at 3 months of age (*n* = 3 for each group). C) Real‐time quantitative PCR analysis determining the mRNA expression of AP‐1 family genes in four groups of mice (*n* = 6 for each group). D) Representative immunoblotting images showing AP‐1 family protein in four groups of mice (*n* = 3 for each group). E) Quantification of (D). F–H) AC16 clones transfected with shRNA‐targeting *SLC25A49* or negative control were treated with Dox for 24 h. AP‐1 family proteins were measured by immunoblotting F), nucleocytoplasmic separation G), and quantified H) (*n* = 3 for each group). I) Real‐time quantitative PCR analysis determining the mRNA expression of AP‐1 family genes in AC16 clones treated with 0.2 mm G6P or control for 24 h (*n* = 3 for each group). J–M) AC16 clones were treated with 0.2 mm G6P or control for 24 h. AP‐1 family proteins were measured by immunoblotting J), nucleocytoplasmic separation L), and quantified K,M) (*n* = 4 for each group). AP‐1, activator protein‐1; G6P, glucose‐6‐phosphate. Data were presented as means ± SD, and analyzed by one‐way ANOVA test. ns, not significant, *P < 0.05, and ***P < 0.001.

Considering the essential function of energy reprogramming in heart failure progression,^[^ ^21^ ^]^ we investigate the specific mechanisms by which *Slc25a49* deficiency‐mediated energy reprogramming drives Dox‐induced cardiomyopathy. Initially, KEGG pathway analysis and Gene Set Enrichment Analysis revealed that differentially expressed genes were highly enriched in the TNF signaling network (**Figure** **4**A). Furthermore, it is established that overexpression of the TNF signaling pathway can activate the AP‐1 family.^[^ ^22^ ^]^ Our results similarly demonstrated a significant upregulation of mRNA and protein expressions of several AP‐1 subunits, including Fos, FosB, Jun, JunB, and JunD, in Dox‐treated *Slc25a49^HKO^* hearts or *SLC25A49^KD^* cardiomyocytes (Figure 4B–F and Figure S3D, Supporting Information). Additionally, the phosphorylation of AP‐1 is crucial for its nuclear localization and transcriptional function, and we observed elevated p‐JUN levels and nuclear translocation following Dox treatment in vitro (Figure 4G,H). Glycolysis inhibitor 2‐Deoxy‐D‐glucose (2‐DG) effectively suppressed AP‐1 expression and rescued Dox‐induced cardiac injury in AC16 cells, a human cardiomyocyte line (Figure S3E–G, Supporting Information). Given the preceding findings, we questioned if G6P, which shown considerable enrichment in energy reprogramming, may function as a crucial metabolic signaling molecule in the regulation of AP‐1. Notably, the addition of G6P to wild‐type AC16 cell line enhanced AP‐1 expression, its phosphorylation, and nuclear translocation, and increased mRNA expression levels of *ANP* and *BNP*. This suggests that the considerable activation of the AP‐1 family by energy reprogramming and contributes to cardiac dysfunction after Dox treatment (Figure 4I–M and Figure S4A, Supporting Information). To conclude, these data reveal that energy reprogramming resulting from *Slc25a49* deletion, in conjunction with Dox treatment, is initiated to modulate AP‐1 phosphorylation and nuclear translocation ability by elevating G6P levels.

### 2.4 G6P–AP‐1 Axis Aggravates Myocardial Injury by Upregulating Sln

**Figure 5:**
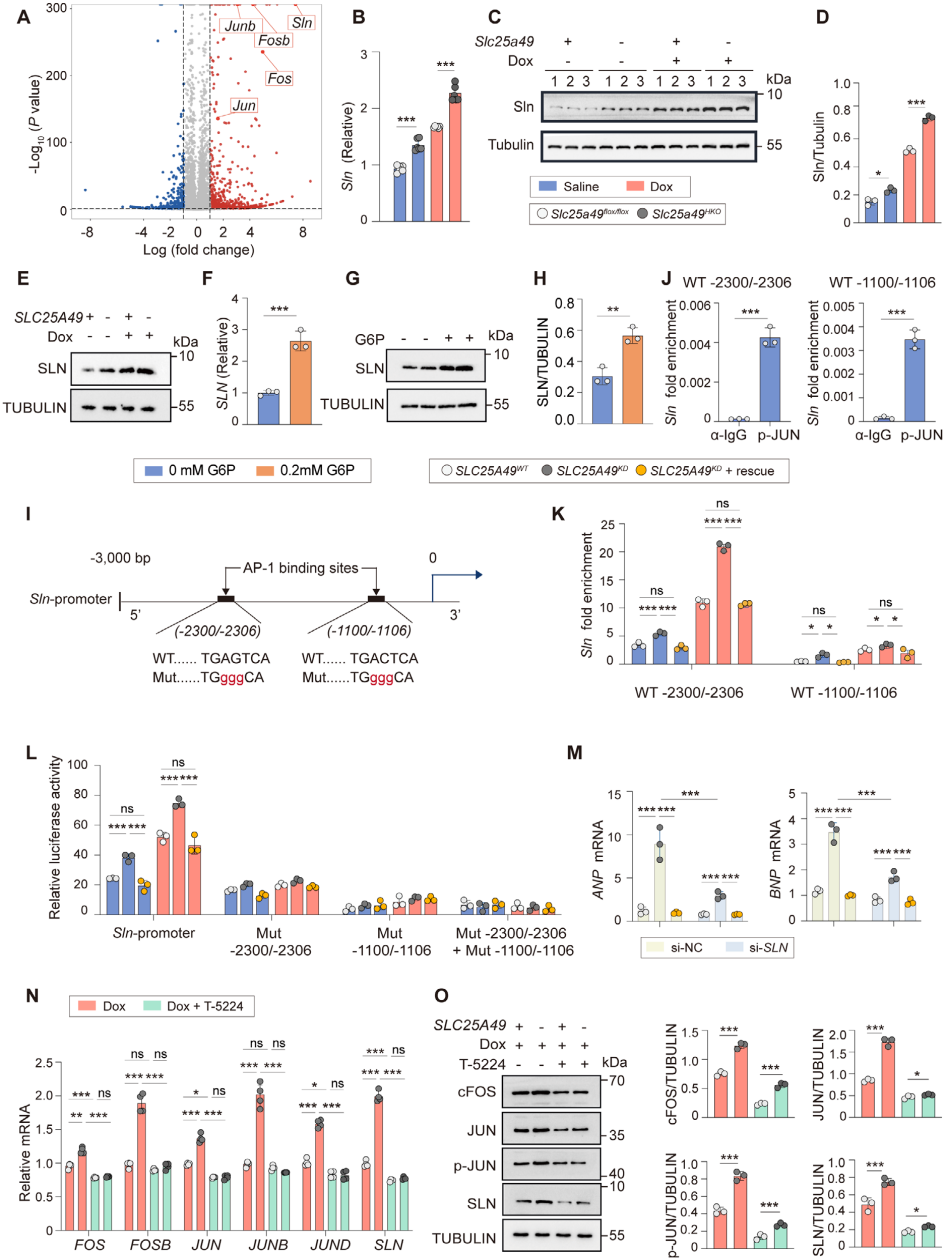
G6P–AP‐1 axis aggravates myocardial injury by targeting and upregulating Sln expression. A) Volcano plot of differentially expressed genes between 3‐month‐old Dox‐treated *Slc25a49^flox/flox^* and *Slc25a49^HKO^* hearts. B) Real‐time quantitative PCR analysis determining the mRNA expression of *Sln* in *Slc25a49^flox/flox^* and *Slc25a49^HKO^* hearts with or without Dox‐treatment at 3 months of age (*n* = 5 for each group). C) Representative immunoblotting images showing Sln protein expression in four groups of mice (*n* = 3 for each group). D) Quantification of (C). E) Representative immunoblotting images showing SLN protein expression in AC16 clones of *SLC25A49^WT^* and *SLC25A49^KD^* with or without Dox‐treatment (*n* = 3 for each group). F) Real‐time quantitative PCR analysis determining the mRNA expression of *SLN* in AC16 clones added with 0.2 mm G6P or a control for 24 h (*n* = 3 for each group). G,H) Representative immunoblotting images showing SLN protein expression in AC16 clones added with 0.2 mm G6P or a control for 24 h, and quantified H) (*n* = 3 for each group). I) In the murine *Sln* promoter, a scheme summarizing the two predicted AP‐1 binding sites and their corresponding mutant forms. WT, wild‐type *Sln* promoter; Mut, *Sln* promoter with mutated AP‐1 binding site (red). J,K) ChIP‐qPCR analysis of the interaction between AP‐1 and two AP‐1 binding sites in the murine *Sln* promoter region under basal condition J) or Dox‐treated condition K). L) As indicated in panel (I), *Sln* promoter luciferase reporter plasmids containing wild‐type (WT) or mutated (Mut) AP‐1 binding sites were transfected into AC16 cell lines, and their luciferase reporter activities were assayed. M) Real‐time quantitative PCR analysis determining the mRNA expression of *ANP* and *BNP* in AC16 clones treated with *SLN* siRNA for 24 h (*n* = 3 for each group). N) Real‐time quantitative PCR analysis determining the mRNA expression of AP‐1 family and *SLN* genes in Dox‐treated AC16 clones added with 40 µm T‐5224 or a control for 24 h (Dox, 500 nm; *n* = 3 for each group). O) Dox‐treated AC16 clones were added with 40 µm T‐5224 or a control for 24 h. AP‐1 family and SLN proteins were measured by immunoblotting and quantified (*n* = 3 for each group). AP‐1, activator protein‐1; Sln, sarcolipin; G6P, glucose‐6‐phosphate. Data were presented as means ± SD, and analyzed by one‐way ANOVA test. ns, not significant, *P < 0.05, **P < 0.01, and ***P < 0.001.

Next, we sought to investigate the role of the G6P–AP‐1 axis in the deterioration of cardiac function and the onset of heart failure. We compared the mRNA expression in RNA‐seq data between Dox‐treated *Slc25a49^HKO^* and *Slc25a49^flox/flox^* mice. We identified that the expression of *Sln* was the most significantly up‐regulated (**Figure** **5**A). Compared with the control, *Slc25a49* deletion remarkably increased Sln mRNA and protein levels in both Dox‐treated mice and AC16 cells (Figure 5B–F). Sln is a recognized inhibitor of the sarco/endoplasmic reticulum Ca^2+^ ATPase (SERCA, which expression is significantly enhanced in cardiac dysfunction disorders.^[^ ^23^ ^]^ Consequently, we inquired if the G6P–AP‐1 axis induces heart dysfunction by regulating *Sln* transcription, which was addressed by demonstrating that G6P addition increased SLN expression in wild‐type AC16 cell lines (Figure 5G,H).

Subsequently, we investigated the transcriptional regulatory effect of the G6P–AP‐1 axis on *SLN*. The interaction between AP‐1 and the predicted AP‐1 binding sites (−2300/−2306 bp, TGAGTCA; −1100/−1106 bp, TGACTCA) in the murine *Sln* promoter region was analyzed (Figure 5I). The interaction between AP‐1 and the two predicted AP‐1 binding sites in the murine *Sln* promoter region was validated by Chromatin immunoprecipitation (ChIP)‐qPCR (Figure 5J). The interaction between AP‐1 and AP‐1 binding sites was augmented in *SLC25A49* knockdown cardiomyocytes subjected to Dox treatment (Figure 5K). To further validate the interaction between AP‐1 and its binding sites, the 3000 bp upstream sequence in the murine *Sln* promoter region containing the two AP‐1 binding sites and corresponding loss‐of‐function mutations were cloned into the luciferase reporter vectors (Figure 5I). The wild‐type murine *Sln* promoter activity was enhanced by Dox in AC16 cell lines, whereas reporters containing mutated AP‐1 binding consensus sequence at either or both AP‐1 binding sites failed to respond to Dox treatment (Figure 5L).

Upon confirming that the G6P–AP‐1 axis enhanced *Sln* expression, we postulated that eliminating *Sln* levels might mitigate Dox‐treated *Slc25a49*‐deletion‐induced cardiomyopathy. To test this hypothesis, AC16 cell lines were transfected with *SLN* siRNA for subsequent investigation (Figure S5A, Supporting Information). *SLN* knockdown substantially mitigated cardiac toxicity generated by Dox in cardiomyocytes, as evidenced by decreased *ANP* and *BNP* mRNA expressions (Figure 5M and Figure S5B, Supporting Information). Moreover, we found that the AP‐1 inhibitor, T‐5224, decreased *SLN* expression in cardiomyocytes, and further knockdown of *SLN* gene could not additionally enhance the protective cardiac effect (Figure 5N,O and Figure S5C, Supporting Information). These data collectively suggest that the lack of *Slc25a49* combined with Dox treatment stimulates the G6P–AP‐1 axis, leading to the upregulation of *Sln* expression and subsequent cardiac dysfunction.

### 2.5 AP‐1 Inhibition Abrogates Dox‐Induced Cardiomyopathy

**Figure 6:**
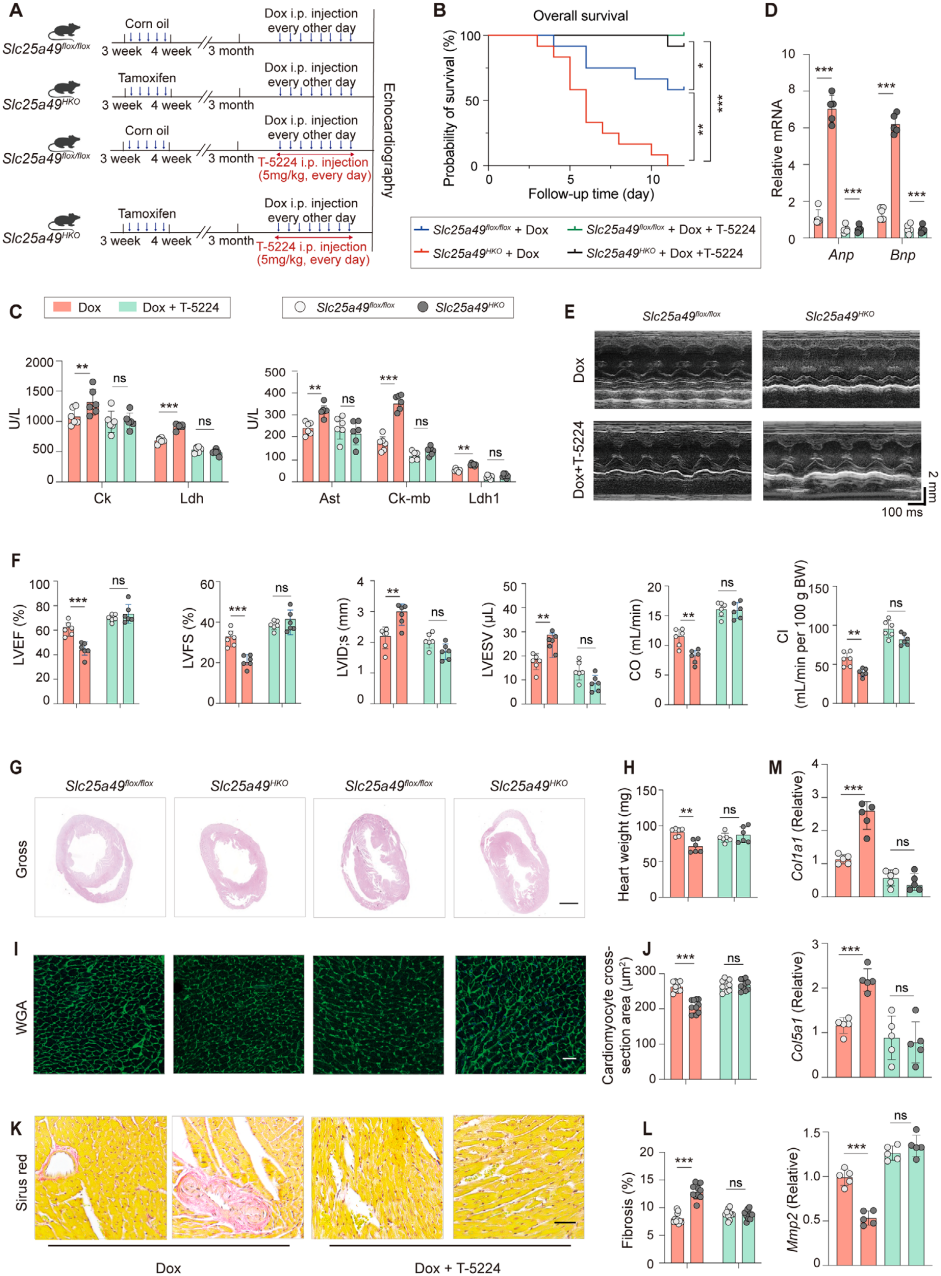
Inhibiting AP‐1 rescues Dox‐induced cardiomyopathy. A) Schematic diagram illustrating the experimental strategy for AP‐1 inhibition. B) Kaplan–Meier plot demonstrating decreased mortality in heart failure mice after AP‐1 inhibition (*n* = 12 for each group). C) Serum levels of Ck, Ldh, Ast, Ck‐mb, and Ldh‐1 were measured in four groups of mice (*n* = 6 for each group). D) Real‐time quantitative PCR analysis of *Anp* and *Bnp* mRNA expression in four groups of mice (*n* = 5 for each group). E) Echocardiography of Dox‐treated *Slc25a49^flox/flox^* and *Slc25a49^HKO^* mice with or without AP‐1 inhibition at 3 months. F) Echocardiography parameters of (E) were calculated (*n* = 6 for each group). G) Horizontal morphology of four groups of mice at 3 months (scale bar = 1 mm). H) Comparison of heart weight in four groups of mice at 3 months (*n* = 6 for each group). I) Wheat germ agglutinin (WGA) staining to compare the sectional cardiomyocyte size in four groups of mice (Scale bar = 100 µm). J) Quantification of cardiomyocyte area in (I) using Image J (*n* = 10 for each group). K) Comparison of Sirus Red staining in four groups of mice (Scale bar = 50 µm). L) Quantification of (K) (*n* = 10 for each group). M) Real‐time quantitative PCR analysis of *Col1a1*, *Col3a1*, and *Mmp2* mRNA expression in four groups of mice (*n* = 5 for each group). Ck, creatine kinase; Ldh, lactic dehydrogenase; Ast, aspartate aminotransferase; Anp, atrial natriuretic peptide; Bnp, brain natriuretic peptide; LVEF, left ventricular ejection fraction; LVFS, left ventricular fraction shortening; LVID;s, left ventricular internal dimension at systole; LVESV, left ventricular end‐systolic volume; CO, cardiac output; and CI, cardiac input. Data were presented as means ± SD, and analyzed by one‐way ANOVA test. ns, not significant, *P < 0.05, **P < 0.01, and ***P < 0.001.

Given the association of aberrant AP‐1 activity with Dox‐induced cardiomyopathy, we examined whether pharmacological suppression of AP‐1 could mitigate Dox‐induced heart dysfunction. Consequently, the AP‐1 inhibitor (T‐5224, 5 mg kg^−1^ IP daily, totaling 80 mg kg^−1^) or corn oil was randomly delivered to 3‐month‐old male *Slc25a49^HKO^* or *Slc25a49^flox/flox^* mice throughout Dox‐induced cardiomyopathy (**Figure** **6**A). The Kaplan–Meier survival analysis demonstrated that T‐5224 medication significantly protected mice from Dox‐induced cardiomyopathy, as indicated by a notable enhancement in survival outcomes post‐T‐5224 intervention (Figure 6B). Decreased levels of myocardial enzyme markers in the serum (Ck, Ck‐mb, Ldh, Ldh‐1, and Ast), and reduced mRNA expression of fetal cardiac genes (*Anp* and *Bnp*) were observed in mice with Dox‐induced cardiomyopathy (Figure 6C,D), indicating a restoration of cardiac function. Echocardiographic analysis showed that T‐5224 administration recovered LVEF, LVFS, CO, CI, LVID;s and LVESV (Figure 6E,F). Histological analysis revealed that AP‐1 suppression mitigated left ventricular dilatation (Figure 6G), heart weight (Figure 6H), cardiomyocyte volume (Figure 6I,J), and fibrosis (Figure 6K,L). Besides, the mRNA expression of myocardial fibrosis‐related genes (*Col1a1*, *Col5a1*, and *Mmp2*) was reinstated in mice administered T‐5224 (Figure 6M). In addition, T‐5224 treatment inhibited Dox‐induced Sln expression in heart tissues, without any effect on the activation of NF‐κB signaling (Figure S6, Supporting Information). Taken together, these findings suggest that pharmacological inhibition of AP‐1 can elicit functional and morphological advantages in heart failure, highlighting the potential therapeutic benefits for cardiomyopathy and heart failure.

## 3 Discussion

This study unveiled multiple noteworthy discoveries. First, *Slc25a49* is essential for sustaining normal mitochondrial activity and energy metabolic equilibrium in cardiomyocytes. *Slc25a49* deficiency is associated with the development of Dox‐induced cardiomyopathy by promoting a metabolic shift from OXPHOS to glycolysis in mitochondria. Second, we identified that a Dox‐induced glycolytic metabolite G6P, which drives the phosphorylation and nuclear translocation of AP‐1, exacerbates myocardial injury via transcriptionally upregulating the expression of sarcoplasmic reticulum calcium importer Sln. Third, the AP‐1 inhibitor T‐5224 demonstrated a considerable protective efficacy against Dox‐induced cardiomyopathy, indicating that AP‐1 may be a viable target for the therapy of this condition.

Our prior research indicated that *Slc25a49* deficiency results in diminished mitochondrial NAD^+^ levels in Hela cells.^[^ ^18^ ^]^ Given that NAD^+^ is an essential coenzyme for several key dehydrogenase reactions in the tricarboxylic acid (TCA) cycle, including isocitrate dehydrogenase, α‐ketoglutarate dehydrogenase, and malate dehydrogenase, a deficiency of this coenzyme results in a reduction in the flux of the TCA cycle.^[^ ^24^ ^]^ In addition, the reduction of the NADH produced by the TCA cycle leads to a further reduction in the supply of electrons to the respiratory chain, hence compromising the efficacy of OXPHOS.^[^ ^25^ ^]^ In this instance, the cellular ATP production is inadequate and the energy supply is constrained, prompting cell to commence energy reprogramming and resort to glycolysis. Previous studies on Dox‐induced cardiomyopathy have consistently demonstrated impaired mitochondrial bioenergetics accompanied by compensatory upregulation of glycolytic flux.^[^ ^26^, ^27^ ^]^ However, our findings reveal a distinctive role of Slc25a49 in this pathological process. Specifically, genetic ablation of *Slc25a49* exacerbates Dox‐induced cardiomyopathy by amplifying the metabolic shift from OXPHOS to glycolysis. This is evidenced by the marked downregulation of mitochondrial ETC genes, impaired OXPHOS capacity, and elevated levels of glycolytic intermediates, particularly G6P, in Dox‐treated *Slc25a49^HKO^* hearts. The upregulation of *Slc25a49* expression in wild‐type mice following Dox treatment may represent an adaptive protective mechanism that maintains energy homeostasis by modulating metabolic pathways, potentially mitigating Dox‐induced adverse effects through the regulation of glycolytic flux. Furthermore, pharmacological inhibition of glycolysis using 2‐DG was found to suppress both *AP‐1* and *SLN* expression at transcriptional and translational levels, subsequently attenuating Dox‐induced cardiomyocyte injury. G6P is a key intermediate in glycolysis, catalyzed by HK‐1. In vitro experiments further demonstrated that G6P could elevate AP‐1 levels, augmenting its phosphorylation and nuclear accumulation. Nonetheless, the fundamental processes by which G6P stimulates AP‐1 warrant further investigation in subsequent investigations.

AP‐1 family consists of transcription factors, typically c‐Jun, JunD, along with members of the Fos and ATF families, which are involved in cellular responses to growth factors, cytokines, neurotransmitters, and other intercellular signaling molecules.^[^ ^28^ ^]^ In cardiac diseases, the activity of AP‐1 is markedly elevated. In patients with myocardial infarction, the expression of AP‐1 and matrix metalloproteinases (MMP) is significantly increased, indicating that AP‐1 may be crucial in ventricular remodeling.^[^ ^29^ ^]^ AP‐1 regulates the transcription of *MMP‐2* in cardiomyocytes, hence facilitating myocardial fibrosis and remodeling. Additionally, AP‐1 exerts a crucial influence on cardiac hypertrophy and apoptosis. Studies have demonstrated that AP‐1 inhibition safeguards cardiomyocytes from hypertrophy and apoptosis.^[^ ^30^ ^]^ In this study, we reveal that a metabolic shift from OXPHOS to glycolysis significantly increase AP‐1 expression in Dox‐treatment hearts. Inhibiting AP‐1 expression by its highly selective inhibitor, T‐5224, corrects impaired cardiac function, showing a broad medicinal clinical value in Dox‐induced myocardial injury.

Our study uncovers for the first time that AP‐1 transcription factor binds to *Sln* and augments its expression, thereby contributing to cardiomyopathy. The function of Sln in heart disease is primarily manifested in its modulation of sarcoplasmic reticulum calcium ions import in cardiomyocytes. Sln is capable of directly binding to SERCA2a, a calcium‐pumping protein whose primary function is to facilitate the transport of intracellular calcium ions back to the sarcoplasmic reticulum, thereby reducing its affinity for Ca^2+^.^[^ ^31^ ^]^ This results in a reduction in Ca^2+^ reuptake and an impact on the contractile capacity of the heart. Upon SLN binding to phospholamban (PLN), a super inhibitory complex is formed, markedly amplifying the inhibitory impact on SERCA2a. This pronounced inhibitory impact is attained by enhancing the binding stability of PLN to SERCA2a.^[^ ^23^ ^]^ Overexpression of *Sln* has been demonstrated to impair cardiac contractility, as evidenced by a reduction in the peak calcium ion transient amplitude and a prolonged decay time in cardiomyocytes.^[^ ^32^ ^]^ We consistently demonstrate that inhibition of *SLN* by siRNA downregulates *ANP* and *BNP* mRNA, alleviating cardiac dysfunction. Excitingly, our study indicates that G6P–AP‐1–Sln axis is a key pathogenic signaling pathway mediating Dox‐induced cardiac dysfunction in both the wildtype and *Slc25a49* knockout mice.

Furthermore, we propose an innovative therapeutic method targeting the G6P–AP‐1–Sln axis to alleviate Dox‐induced cardiotoxicity. HK1 is a crucial enzyme that promotes the conversion of glucose into G6P.^[^ ^33^ ^]^ Inhibiting HK1 activity reduces G6P synthesis, hence curtailing excessive glycolytic activation. Regrettably, there is presently an absence of effective inhibitors for HK1. The AP‐1 inhibitor T‐5224 exhibited considerable protective effects against Dox‐induced cardiotoxicity by inhibiting the central mediating function of AP‐1. Further modification of its specificity and pharmacokinetic characteristics is essential to improve its efficacy and safety for clinical use. Besides directly blocking AP‐1, modulating its downstream signaling pathway and specific targets is a feasible method. In this study, *Sln* was identified as a key target of AP‐1, and reducing *SLN* expression in vitro mitigated Dox‐induced cardiomyocyte injury, thus potentially alleviating Dox‐induced cardiotoxicity. In therapeutic settings, gene therapy techniques such antisense oligonucleotides or RNA interference can be employed to selectively reduce *SLN* expression. When regulating SLN expression, it is crucial to carefully manage its expression level to prevent potential detrimental effects on the normal calcium homeostasis regulatory mechanisms of cardiomyocytes resulting from excessive SLN inhibition.^[^ ^34^ ^]^

Nevertheless, several limitations should be acknowledged in this study. The first pertains to potential gender‐related bias in our experimental design. Considering the documented interactions between Dox and estrogen‐mediated pathways, along with other female‐specific hormonal factors, male mice exhibit greater susceptibility to Dox‐induced cardiotoxicity compared to their female counterparts.^[^ ^35^ ^]^ Consequently, we exclusively employed male mouse models throughout this study, which may limit the generalizability of our findings across both genders. Second, while our investigation has primarily focused on *Sln*, the potential contributions of other pathways identified in our RNA‐seq analysis warrant further consideration. For instance, a broad downregulation of mitochondrial respiratory ETC genes in Dox‐treated *Slc25a49^HKO^* hearts was observed, indicating a significant disruption in mitochondrial energy metabolism. Additionally, genes related to the TNF signaling network were also highly enriched among the differentially expressed genes, which may affect other downstream effectors contributing to cardiac dysfunction. Furthermore, we identified substantial alterations in genes regulating extracellular matrix (ECM) remodeling, including those encoding collagen‐associated proteins and MMPs. Targeting these genes might potentially slow down or reverse the fibrotic process in the heart. Third, the current study concerned exclusively with chronic Dox‐induced cardiomyopathy, and did not address the effect of *Slc25a49* on acute Dox‐induced cardiomyopathy. Acute models typically cause rapid and severe cardiac damage, which may mainly reflect the immediate cytotoxic effects of Dox. Compared to acute models where a single high‐dose injection of Dox is used, chronic model provides a more comprehensive view of the long‐term effects. However, acute Dox‐induced cardiomyopathy is also clinically significant, and it is anticipated that subsequent studies will investigate the role of *Slc25a49* in this condition, which will contribute to a more comprehensive understanding of the mechanisms of Dox‐induced cardiotoxicity.

## 4 Conclusions

Our study reveals that *Slc25a49*‐mediated energy reprogramming serves as a key regulator of cardiomyocyte remodeling and dysfunction induced by Dox. To our best knowledge, this is the first study to provide a theoretical foundation for further investigation of therapeutic approaches targeting G6P–AP‐1–Sln axis in Dox‐induced cardiomyopathy. In particular, we identified that the AP‐1 inhibitor T‐5224 exhibits an effective protection against Dox‐induced cardiomyopathy. Therefore, given the profound clinical significance of Dox and mitochondrial bioenergetics, our findings not only expand the molecular mechanism of Dox‐induced cardiomyopathy but also provide novel insights into potential therapeutic targets.

## 5 Experimental Section

### Animal Model

Mice with *Slc25a49* floxed alleles (*Slc25a49* ^flox/flox^; C57BL/6J background; GemPharmatech, Nanjing, China; catalog no. T018164) were bred with *Myh6‐Cre/ERT2* transgenic mice (C57BL/6J background; GemPharmatech, Nanjing, China; catalog no. T004858) to generate the cardiomyocyte‐specific *Slc25a49* gene inducible knockout mice (*Slc25a49* ^HKO^). Animals were housed in a specific pathogen‐free facility with access to standard mouse chow and water and were exposed to an alternating 12‐h light/12‐h dark cycle in controlled temperature conditions. The investigation conforms to the Guide for the Care and Use of Laboratory Animals published by the US National Institutes of Health (NIH Publication No. 85‐23, revised 1985). All animal experiments received approval and were performed in compliance with the guidelines approved by the China Agricultural University ethics committee (approval number: AW50804202‐5‐5).

To obtain cardiac‐specific *Slc25a49* knockout mice (*Slc25a49* ^HKO^) or control mice (*Slc25a49* ^flox/flox^), tamoxifen or corn oil was intraperitoneal injected (75 mg kg^−1^) to *Slc25a49* ^flox/flox^; *Myh6‐Cre/ERT2* mice for 5 consecutive days at 3 weeks of age. 12‐week‐old male *Slc25a49* ^flox/flox^ and *Slc25a49* ^HKO^ mice were randomly divided into the following groups: *Slc25a49* ^flox/flox^ + Saline group, *Slc25a49* ^flox/flox^ + Dox group, *Slc25a49* ^HKO^ + Saline group, *Slc25a49* ^HKO^ + Dox group. Mice received intraperitoneal injections of Dox or saline at equivalent dosages every other day for 16 days, resulting in a cumulative dose of 24 mg kg^−1^ of Dox.^[^ ^19^ ^]^ To validate the therapeutic efficacy of T‐5224 in Dox‐induced cardiomyopathy, 12‐week‐old male Dox‐treated *Slc25a49* ^flox/flox^ and *Slc25a49* ^HKO^ mice were randomized into the following groups: *Slc25a49* ^flox/flox^ + Dox group, *Slc25a49* ^flox/flox^ + Dox + T‐5224 group, *Slc25a49* ^HKO^ + Dox group, *Slc25a49* ^HKO^ + Dox + T‐5224 group. Mice were administered Dox intraperitoneally, either alone or in conjunction with T‐5224. Dox injections were administered every other day for a total of 8 injections over a period of 16 days, resulting in a cumulative dose of 24 mg kg^−1^, while T‐5224 intraperitoneal injections were given daily starting on the same day as Dox injections, totaling 16 injections and culminating in a cumulative dose of 80 mg kg^−1^. Following echocardiographic evaluation, mice were sacrificed for subsequent experiments. Mice were euthanized using 2% inhaled isoflurane followed by cervical dislocation.

### Echocardiogram Evaluation

Mouse cardiac geometry and function were examined using a Vevo 3100 High‐Resolution in Vivo Micro‐Imaging System (FUJIFILM VisualSonics, Toronto, ON, Canada). Mice were anesthetized with 1.5% isoflurane and positioned supine on a heating table. The heart rates were maintained at 450–500 beats per minute (bpm), whenever possible. Left ventricle (LV) echocardiography is evaluated in parasternal long‐axis and short‐axis views at a frame rate of 233 Hz. LVID, LVESV, FS, EF, and CO were calculated from the left ventricular dimensions at the end of systole and diastole. The CI (mL min^−1^ per 100 g body weight) is the CO (mL min^−1^) normalized to 100 g body weight. All the studies and analyses were performed blind to cardiac status.

### Measurement of Serum Myocardial Enzymes

Whole blood samples were placed at room temperature for 2 h, then centrifuged at 3000 rpm for 15 min at 2–8 °C, and the supernatants were taken as serum. Serum Ast, Ck, and Ldh were measured using kits (S03040, S03024, S03034, Rayto, Shenzhen, China) respectively. Serum Ldh‐1 and Ck‐mb were measured using the kits (C058‐e, C060, Changchun Huili, Changchun, China).

### Histological Analysis

After echocardiogram evaluation, hearts were perfused with chilled 0.1 m PBS (pH 7.4), fixed overnight in 4% paraformaldehyde (pH 7.4), embedded in paraffin, and serially sectioned at 5 µm thickness. Sections were stained with hematoxylin and eosin (H&E) (G1120, Solarbio, China) and underwent routine histological examination with an optical microscope. Sections were stained with Sirius Red (G1472, Solarbio, China) to measure collagen deposition. Cardiomyocyte cross‐sectional size was assessed by WGA staining (W11261, Invitrogen, CA). Acquisition of histological images was carried out with a light microscope (Zeiss Axioplan2). Three adjacent sections from each mouse were quantified using ImageJ software (version 1.52, National Institutes of Health). In the case of the pathological stained images, in order to guarantee the originality and authenticity of the images, no adjustments were made to parameters, such as contrast and brightness following image acquisition.

### Transmission Electron Microscopy

Hearts were sectioned into 1–2 mm^3^ pieces, and fixed for 2 h at room temperature in 2.5% glutaraldehyde and 0.1 m sodium cacodylate buffer. After being cooled to 4 °C overnight, the collected samples were embedded, ultramicro dissected to obtain 90‐nm thin sections, and captured using a Hitachi HT7800 transmission electron microscope (Hitachi, Japan). All images were captured with a slow‐scan 2k CCD (charge‐coupled device) camera.

### Cell Culture and Treatments

Human AC16 cardiomyoblasts (CL‐0790) purchased from Pricella Biotechnology (Wuhan, China) were used in this study. Lentiviral vectors encoding shRNA targeting *SLC25A49* were purchased from Hanhen Biotechnology (Shanghai, China) (Table S1, Supporting Information), and *SLC25A49* knockdown clones (*SLC25A4^KD^*) were established as described.^[^ ^18^ ^]^

### RNA Extraction and Real‐Time Quantitative PCR (RT‐qPCR)

Total RNA was isolated from cardiomyocytes or hearts using TRIzol (15596026CN, Thermo, USA), and reverse‐transcribed into cDNA using Reverse Transcription Reagent kits (FT301, Vazyme, China). RT‐qPCR analysis was performed on an Applied Biosystems StepOnePlus real‐time PCR instrument (ABI 7500) combined with SYBR Green PCR mix (Q711‐02, Vazyme, China) in accordance with the manufacturer's instructions. All reactions were performed in triplicate, and specificity was monitored using melting curve analysis. Results were analyzed using the comparative CT method. Samples were normalized to *β‐actin* to account for cDNA loading differences. The primer sequences for PCR used were summarized in Table S2 (Supporting Information).

### Immunoblotting

Cultured cardiomyocytes and cardiac tissue were homogenized in RIPA buffer containing protease inhibitors. The homogenate was cleared by centrifugating at 4 °C for 15 min at 12 000 rpm, and the supernatant (containing the protein fraction) was collected. For immunoblotting experiments, 40–80 µg of cleared lysate were separated by 10% SDS‐polyacrylamide gel electrophoresis and transferred to nitrocellulose membranes. The membranes were blocked with 5% milk at room temperature for 2 h and probed with specific antibodies overnight at 4 °C, followed by probed with horseradish peroxidase‐conjugated antimouse or antirabbit secondary antibodies at room temperature for 2 h. All immunoblots were carried out using a chemiluminescence reagent (185 001, Tanon, Shanghai, China) and the signals were collected using ChemiScope3600MINI (Clinx Science Instruments, China). During the experiment, no alterations were made to any of the parameters except for the implementation of chemiluminescent detection, and the instrument was automatically exposed to ensure the objectivity and accuracy of the results.

### Bulk RNA Sequencing (RNA‐seq)

Total RNA was extracted from mouse hearts using TRIzol reagent, reverse transcribed and amplified following standard procedures. BGISEQ‐500 platform (BGI, Wuhan, China) was used to construct sequencing libraries. SOAPnuke was used to filter the sequencing data to remove reads containing sequencing aptamers. The unadulterated reads were preserved in FASTQ format. HISAT2 and STAR aligner were used to map the transcribed reads to the mouse genome (GRCm38), and ENSEMBL transcripts were counted. The identification of differentially expressed genes (DEGs) involved calculating and analyzing the expression of all mapped genes using DESeq2, based on their corresponding fragments per kilobase per million (FPKM) values. A false discovery rate threshold of 0.05 was employed to evaluate meaningful differences. Both bioinformatics and significance analyses were performed in triplicate. BGI Genomics executed a systematic and uniform protocol for all RNA‐seq analysis processes, encompassing RNA extraction, quantification, and cDNA library preparation.

For functional analysis, DEGs were analyzed for KEGG (https://www.kegg.jp/) enrichment using Phyper based on the hypergeometric test. Statistical differences between enriched terms and pathways were corrected using the Bonferroni test with a strict Q threshold of ≤ 0.05. Genes associated with changes in *Slc25a49* knockout hearts in Dox‐induced cardiotoxicity were collected and displayed as volcano plots in Rstudio using the ggplot2 package. The mitochondrial electron microparticle mapping data were processed in Rstudio with the Pheatmap package, which generated heat maps depicting the transcript levels of mitochondrial ETC complex subunits (Com I∼V) and the AP‐1 family (*Fos, FosB, Jun, JunB*, and *JunD*) from the RNA‐seq dataset.

### Detection of ATP Level

Cardiac tissue and cellular ATP levels were determined by the Enhanced ATP Assay Kit (S0027, Beyotime, China) following the manufacturer's protocol.

### Measurement of Mitochondrial Membrane Potential

Mitochondrial membrane potential was determined using the Mitochondrial Membrane Potential Assay Kit (TMRE) (C2001S, Beyotime, China).

### Cell Bioenergetic Profiling

Mitochondrial respiratory capacity was determined by measuring oxygen consumption rate (OCR) and extracellular acidification rate (ECAR) in real time using a Seahorse XFe 96 extracellular flux analyzer (Seahorse Biosciences, Agilent Technologies, USA). The human cardiomyocyte cell line AC16 was inoculated at 5 × 10^3^ cells per well into assay microtiter wells of 10% DMEM/F12. Subsequent to targeted stimulation, the medium was substituted with Seahorse XF DMEM buffer (containing 10 mm glucose, 2 mm glutamine, and 1 mm pyruvate) and then incubated for 1 h at 37 °C in a CO_2_‐free incubator. The plates were then injected sequentially with the following compounds in the OCR experiment: Oligomycin (ATP synthase inhibitor, 1.5 mm); FCCP (mitochondrial uncoupler, 3 mm); Rot/AA (Complex III and Complex I Inhibitor, 0.5 mm; 103015‐100, XF Cell Mitochondrial Stress Test Kit, Agilent Technologies, USA). Respiratory parameters, including basal respiration, ATP generation‐coupled respiration, maximal respiration, and residual respiratory capacity, were quantified in real time by calculating the average respiratory rate and subtracting the rate before and after compound injection. For the ECAR experiment, the test consisted of four consecutive stages: basal (without drug), glycolysis induction (10 mm glucose), maximal glycolysis induction (1 µm oligomycin), and glycolysis inhibition (50 mm 2DG). The OCR and ECAR in each microwell was normalized to the protein content in each well.

### Metabolomics Test

For metabolomics testing of mouse hearts, tissues were homogenized and lysed by ultrasound (30 000 Hz) in precooled phosphate buffer solution (PBS). Proteins in the supernatant were harvested as metabolite‐free proteomes after repeated centrifugation (1000 g) with 85% cold acetone. Chromatography was performed using liquid chromatography‐mass spectrometry (LC‐MS) (Thermo Fisher Scientific, UltiMate 3000 LC, Orbitrap Elite). Separations were performed on a Hypergod C18 column (100 mm × 4.6 mm, 3 mm particle size) at a flow rate of 0.3 mL min^−1^ followed by a linear gradient separation on an analytical column (Acclaim PepMap C18, 75 mm × 15 cm). The analytical column was heated to 40 °C using an AgileSleeve column heater (Analytical Sales & Service, Inc.), equilibrated with 98% mobile phase A (0.1% formic acid/3% acetonitrile) and 2% mobile phase B (0.1% formic acid/90% acetonitrile), and a constant column flow of 0.3 mL min^−1^ was maintained. Samples were analyzed using SIEVE software (Thermo Fisher Scientific).

### Measurement of Metabolic State

Cardiac tissue and cardiomyocytes were lysed with RIPA buffer. Subsequently, the levels of G6P and lactic acid were determined, respectively, using the G6P Assay Kit with WST‐8 (S0185, Beyotime, China) and Lactic Acid (LA) Content Assay Kit (BC2235, Solarbio, China) in accordance with the manufacturer's protocol.

### Fraction Extraction

Nuclear and cytoplasmic proteins were extracted according to the method recommended by the manufacturer (P0028, Beyotime, Shanghai, China). The human cardiomyocyte cell line AC16 was washed twice with cold 0.1 m PBS (pH 7.4) and centrifuged at 1000 rpm for 5 min before collection in PBS. The cell pellet was resuspended in 200 µL of cytoplasmic extraction reagent A (with 1 mm PMSF added), thoroughly vortexed for 5 s, and lysed on ice for 15 min. Subsequently, 10 µL of cytoplasmic extraction reagent B was added to the cell pellet and homogenized by centrifugation (13 000 rpm, 4 °C, 5 min) to collect the cell membrane fraction. A 50 µL homogenate of Nuclear Extraction Reagent with PMSF was meticulously agitated for 30 min, followed by centrifugation at 13 000 rpm and 4 °C for 10 min to isolate the precipitate, including nuclei. The supernatant was obtained as a nuclear fraction. The amounts of cytoplasmic and nuclear proteins were quantified utilizing the BCA technique.

### ChIP and PCR

Specific protein‐DNA interactions were identified using ChIP in conjunction with qPCR (Chromatin Immunoprecipitation Assay Kit, 17‐295, Millipore, USA). The primer sequences for PCR used were listed in Table S2 (Supporting Information).

### Dual‐Luciferase Reporter Assay

AC16 cells at 50% confluence in a 24‐well plate were transfected with 0.1 µg of either wild‐type (WT) or mutant (Mut) luciferase reporter plasmid, which expresses firefly luciferase, together with a renilla luciferase reporter plasmid (pMCS‐Fluc‐SV40‐hRluc‐Neo). WT plasmids incorporated two *Sln* promoter fragments (−2300/−2306 bp; −1100/−1106 bp), while Mut plasmids modified the nucleotide sequence at the AP‐1 binding region of the *Sln* gene promoter. Following transfection, cells were harvested and evaluated utilizing the Dual‐Luciferase Reporter Gene Assay Kit (11402ES60, Shanghai Yeison Biotechnology Co., Ltd.), and autoluminescence variations in each group were quantified using a microtiter plate reader (BioWells, Inc.).

### Statistics Analysis

All statistical calculations were analyzed using GraphPad Prism 10 software, and all summary data are presented as mean ± standard deviation (SD). All experiments were performed at least in triplicate. Student's *t*‐tests were employed to compare the two scenarios. Analysis of variance (ANOVA) and Sidak's multiple comparison test were employed to compare means among more than two groups, contingent upon the data being normally distributed and the assumption of homogeneity of variance being satisfied. The nonparametric Kruskal–Wallis test and Dunn's multiple comparison test were employed for data that do not follow a normal distribution. *P* < 0.05 was deemed statistically significant, with the following designations: *P < 0.05, **P < 0.01, and ***P < 0.001.

## Conflict of Interest

The authors declare no conflict of interest.

## Author Contributions

J.L., Y.L., and P.A. conceived the study. S.W., J.Q., Y.X., C.F., T.X., X.Z., J.S., C.W., Y.C., D.Z., R.L., Y.Z., C.C., and D.J. performed the animal and cell experiments. S.W., J.Q., Y.X., C.F., T.X., and X.Z. analyzed the data. J.L., Y.L., P.A., and S.W. drafted manuscript; and J.L. and S.W. revised the manuscript.

## Supporting information



Supporting Information

## Data Availability

The data that support the findings of this study are available from the corresponding author upon reasonable request.
