# Supplementary material for: Cardiac Slc25a49‐Mediated Energy Reprogramming Governs Doxorubicin‐Induced Cardiomyopathy through the G6P–AP‐1–Sln Axis
Source: Adv Sci (Weinh). 2025 Apr 4;12(26):2502163. doi: 10.1002/advs.202502163 (PMC12244504; doi:10.1002/advs.202502163)
Supplement: Supplementary file 1 — Supporting Information [file ADVS-12-2502163-s001.docx]

**Cardiac *Slc25a49*-Mediated Energy Reprogramming Governs Doxorubicin-Induced Cardiomyopathy Through the G6P–AP-1–Sln Axis**

**Short title: G6P–AP-1–Sln axis in Dox-Induced Cardiomyopathy**

Sitong Wan^1^, Jingyi Qi^1^, Yi Xia^1^, Chang Fan^1^, Teng Xu^2^, Xu Zhang^1^, Jiaxin Shi^1^, Chenxuan Wang^1^, Yitong Cheng^1^, Dongyuan Zhang^1^, Rong Liu^1^, Yinhua Zhu^1^, Changchang Cao ^3^, Dekui Jin ^4^, Peng An^1,^*, Yongting Luo^1,^*, Junjie Luo^1,^*

^1^ Key Laboratory of Precision Nutrition and Food Quality, Department of Nutrition and Health, China Agricultural University, Beijing, 100193, China

^2^ Food Science Institute, Zhejiang Academy of Agricultural Sciences, Hangzhou, 310021, China

^3^ State Key Laboratory of Cardiovascular Disease, Fuwai Hospital, National Center for Cardiovascular Diseases, Chinese Academy of Medical Sciences and Peking Union Medical College, Beijing, 100037, China

^4^ Department of General Practice, The Third Medical Center of Chinese PLA General Hospital, Beijing 100039, China

*Correspondence: luojj@cau.edu.cn (J. Luo), luo.yongting@cau.edu.cn (Y. Luo), an-peng@cau.edu.cn (P. An).

**Supplementary methods**

**Antibodies and reagents**

The following antibodies were used in this study: anti-FOS (1:1000 dilution, 3270S, CST, USA), anti-JUN (1:1000 dilution, ab280089, Abcam, UK), anti-p-JUN (1:1000 dilution, 3270S, CST, USA), anti-SLN (1:1000 dilution, 18395-1-AP, Proteintech, USA), anti-HK1 (1:1000 dilution, A0533, Abclonal, USA), anti-HK2 (1:1000 dilution, A20829, Abclonal, USA), anti-GPI (1:1000 dilution, A6916, Abclonal, USA), anti-P65 (1:1000 dilution, 8242T, Cell Signaling Technology, USA), anti-IkB (1:1000 dilution, 9242S, Cell Signaling Technology, USA), anti-p-P65 (1:1000 dilution, 3033T, Cell Signaling Technology, USA), anti-Histone (1:1000 dilution, 17168-1-AP, Proteintech, USA), anti-β-Tubulin (1:5000 dilution, AC021, Abclonal, USA), Alexa Flour 488 goat anti-mouse IgG (1:200 dilution, ab150113, Abcam, UK), Alexa Fluor 594 goat anti-rabbit IgG (1:200 dilution, ab150080, Abcam, UK).

Tamoxifen (HY-13757A) was purchased from MCE (Shanghai, China) and dissolved in 10 mL corn oil to get a 7.5 mg/kg stock, stored at 4℃. Doxorubicin hydrochloride (D8740) was purchased from Solarbio (Beijing, China) and dissolved in 2.5 mL of water to get a 10 mg/mL stock, stored at -20℃. T-5224 (HY-12270), rotenone (HY-B1756) and 2-Deoxy-D-glucose (HY-13966) were purchased from MCE.

**Cell culture and treatments**

Human AC16 cardiomyoblasts (CL-0790) were purchased from Pricella Biotechnology (Wuhan, China), maintained in DMEM/F12 (PM150310, Pricella, China), supplemented with 10% FBS, and were incubated in a humidified atmosphere with 5% CO_2_ and 95% air at 37 °C. All culture media were supplemented with penicillin (100 U/mL) and streptomycin (100 μg/mL).

Lentiviral vectors encoding shRNA targeting *SLC25A49* were purchased from Hanhen Biotechnology (Shanghai, China) (Table S1), and *SLC25A49* knockdown clones (*SLC25A49^KD^*) were established as described^1^. Briefly, lentiviral vectors were transfected into AC16 cells with Lipofectamine 3000 transfection reagent (L3000015, Thermo FisherScientific, USA). 48 hours after transfection, GFP-positive cells were screened with puromycin. *SLC25A49* gene amplification was performed on the culture clones, and the decrease of *SLC25A49* gene expression by more than 60% signified the successful establishment of the knockdown clone. The plasmid pEnCMV- SLC25A49 -Linker-EGFP-SV40-Neo was purchased from Miao Ling Plasmid Sharing Platform (Wuhan, China), and transfected into *SLC24A49^KD^* cells by Lipofectamine 3000 transfection reagent to rescue the *SLC25A49* level and the increase of *SLC25A49* gene expression by more than 60% signified the successful establishment of the resuce clone (*SLC24A49^KD^* + rescue). To knockdown *SLN* in vitro, AC16 cells were transfected with 25 nM of *SLN* siRNA (SYNBIO, Suzhou, China) or control siRNA using Lipofectamine 3000 transfection reagent (Table S1).

Other reagents for cell treatment were as follows: Dox (500 nM for 24 hours); Rotenone (1 nM for 24 hours); G6P (0.2 mM for 24 hours); T-5224 (40 μM for 12 hours).

**Detection of ATP level**

Cardiac tissue and cellular ATP levels were determined by the Enhanced ATP Assay Kit (S0027, Beyotime, China) following the manufacturer’s protocol. Briefly, cardiac tissues and cardiomyocytes were lysed using ATP lysate buffer and then centrifuged at 12,000 rpm for 5 min at 4°C. The supernatant was harvested to assess the ATP concentration. Relative concentrations of ATP were measured using a luminescence meter (BioTek Instruments, UK) and normalized by protein concentration (BCA Protein Assay Kit, CW0014S, CWBIO, China).

**Measurement of mitochondrial membrane potential**

Mitochondrial membrane potential was determined using the Mitochondrial Membrane Potential Assay Kit (TMRE) (C2001S, Beyotime, China). Briefly, 1 mL of TMRE staining working solution was incorporated into the culture medium, thoroughly mixed, and thereafter incubated in a 37°C cell culture incubator for 30 min. Upon completion of incubation, the supernatant was discarded, the cells were rinsed twice with TMRE staining buffer, and 2 mL of medium was introduced. The assay was performed by flow cytometry (CytoFLEX LX, Beckman Coulter, USA). TMRE was detected at an excitation wavelength of 550 nm and an emission wavelength of 575 nm.

**Chromatin Immunoprecipitation (ChIP) and PCR**

Specific protein-DNA interactions were identified using ChIP in conjunction with qPCR (Chromatin Immunoprecipitation Assay Kit, 17-295, Millipore, USA). Protein-DNA cross-linking was executed by fixing cells in 1% formaldehyde for 10 min at room temperature. DNA-protein complexes from 2 × 10^6^ cells were clipped to a length of 200 to 500 base pairs utilizing an ultrasonicator. Pre-cleared fragments were incubated overnight with 10 μg of p-JUN specific antibody or IgG (as a negative control) and then immunoprecipitated with protein A. Heating overnight at 65°C reversed cross-linking, followed by digestion with proteinase K for 2 h at 45°C. The DNA was subsequently purified using the QIAquick PCR Purification Kit (28104, Qiagen, Germany), followed by qPCR to verify affinity for specific promoter regions. qPCR was performed using primers for specific promoters flanking the p-JUN binding site. The primer sequences for PCR used were listed in Table S2.

**Reference**

1. Wang X, Ji Y, Qi J, Zhou S, Wan S, Fan C, Gu Z, An P, Luo Y, Luo J. Mitochondrial carrier 1 (MTCH1) governs ferroptosis by triggering the FoxO1-GPX4 axis-mediated retrograde signaling in cervical cancer cells. *Cell Death Dis* 2023;**14**:508.


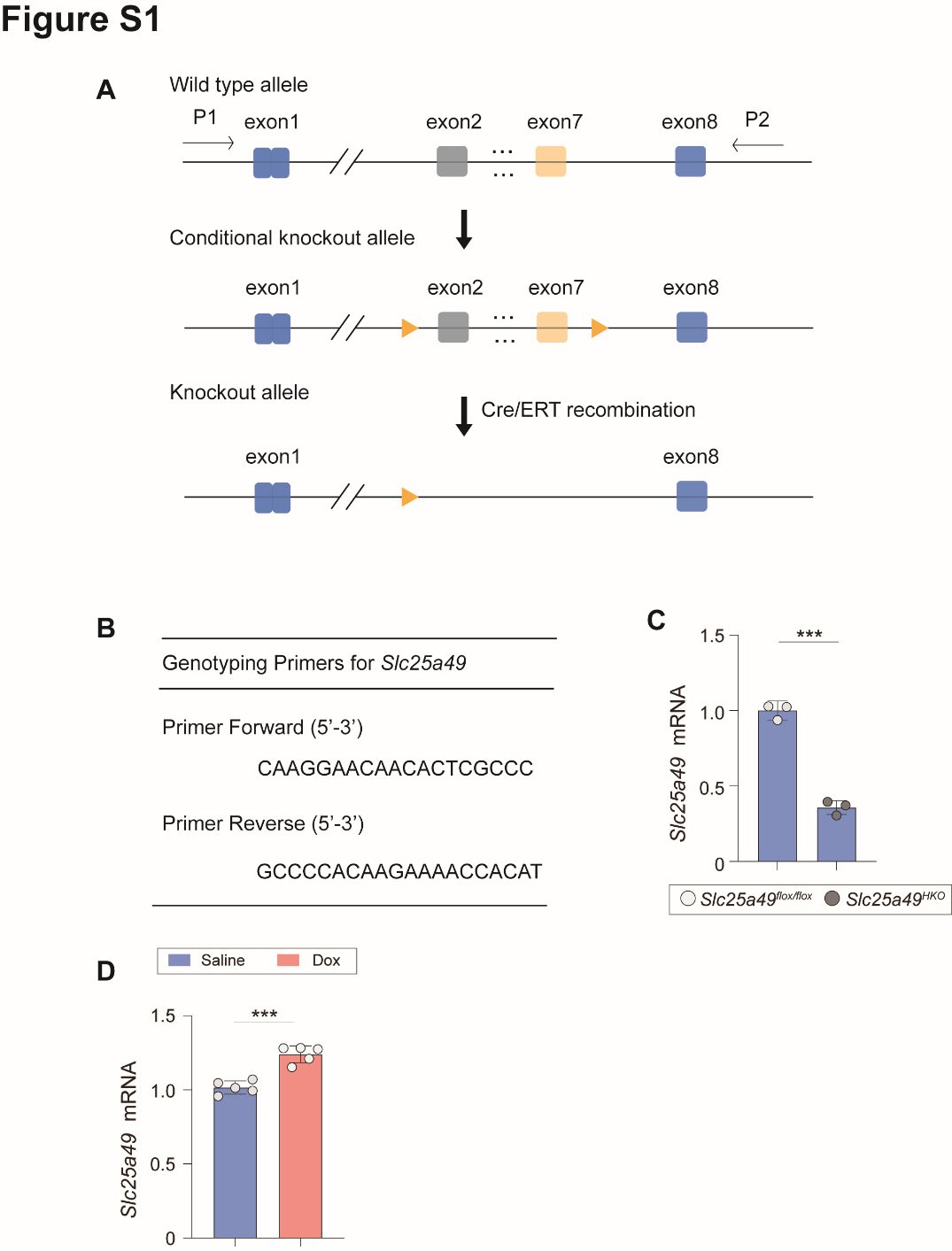


**Figure S1. Construction of cardiac-specific *Slc25a49* knockout mice.** (A) Schematic diagram depicting the strategy used to generate Cre-inducible cardiac-specific *Slc25a49* knockout mice. (B) The primer pair used for genotyping of *Slc25a49^HKO^* mice. (C) *Slc25a49^HKO^* mice and its *Slc25a49^flox/flox^* littermates were screened by reverse transcription-PCR (n = 3 for each group). (D) Real-time quantitative PCR analysis of *Slc25a49* mRNA expression in *Slc25a49^flox/flox^* mice treated with Corn oil or Dox at 3 months (n = 5 for each group). Data were presented as means ± SD, and analyzed by t test. ****P* < 0.001.


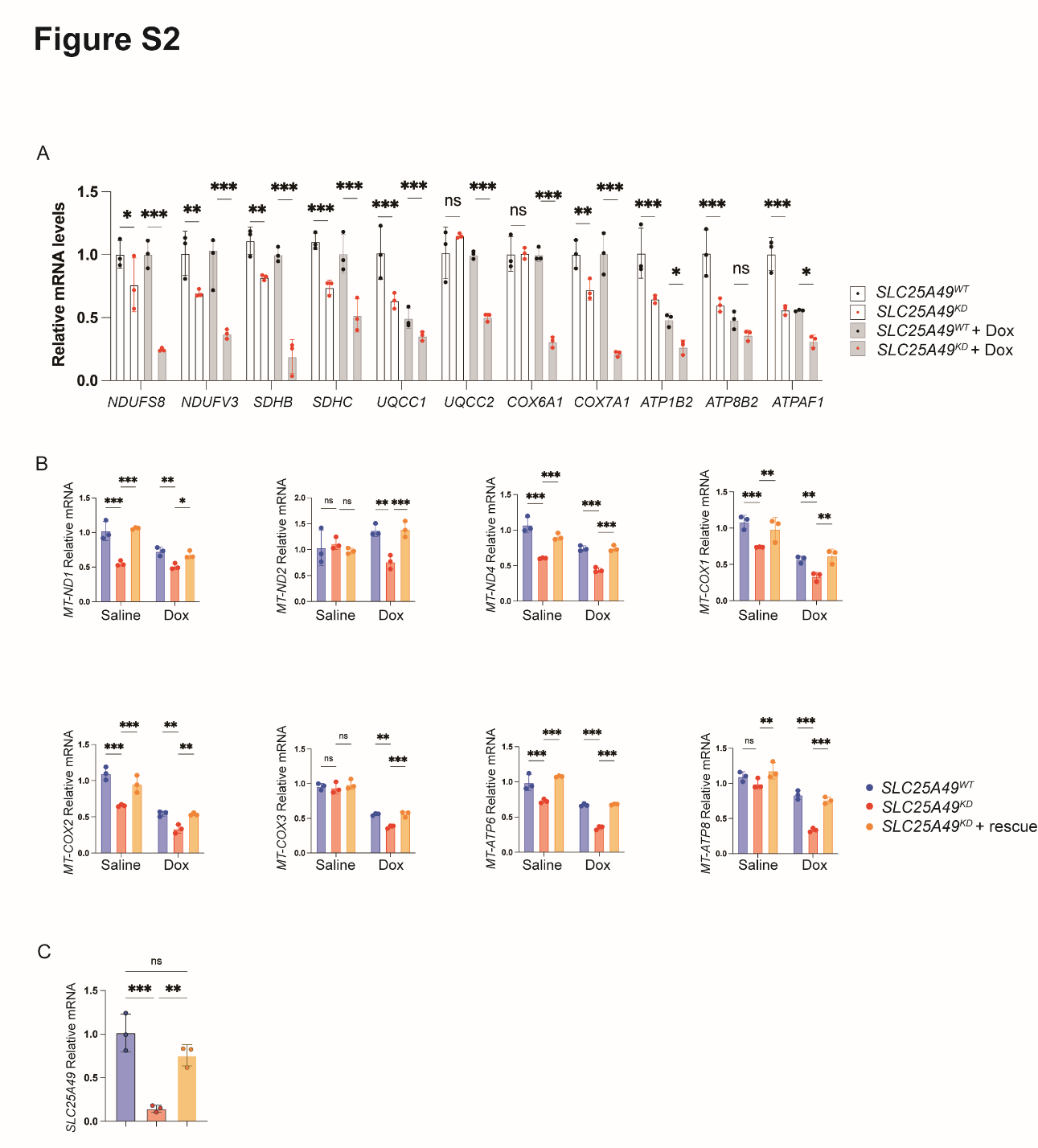


**Figure S2. *SLC25A49^KD^* inhibits OXPHOS in human cardiomyocytes.** (A) Real-time quantitative PCR analysis determining the mRNA expression of selected electron transport chain complex related genes in AC16 clones of *SLC25A49^WT^* and *SLC25A49^KD^* treated with or without Dox for 24 hours (Dox, 500 nM; n = 3 for each group). (B) Real-time quantitative PCR analysis determining the mRNA expression of selected mitochondrial-encoded genes in AC16 clones of *SLC25A49^WT^*, *SLC25A49^KD^*, and *SLC25A49^KD^* + rescue treated with or without Dox for 24 hours (Dox, 500 nM; n = 3 for each group). (C) Real-time quantitative PCR analysis determining the mRNA expression of *SLC25A49* in AC16 of *SLC25A49^WT^*, *SLC25A49^KD^*, and *SLC25A49^KD^* + rescue (n = 3 for each group). Data were presented as means ± SD, and analyzed by one-way ANOVA test. ns, not significant, **P* < 0.05, ***P* < 0.01, and ****P* < 0.001.

**
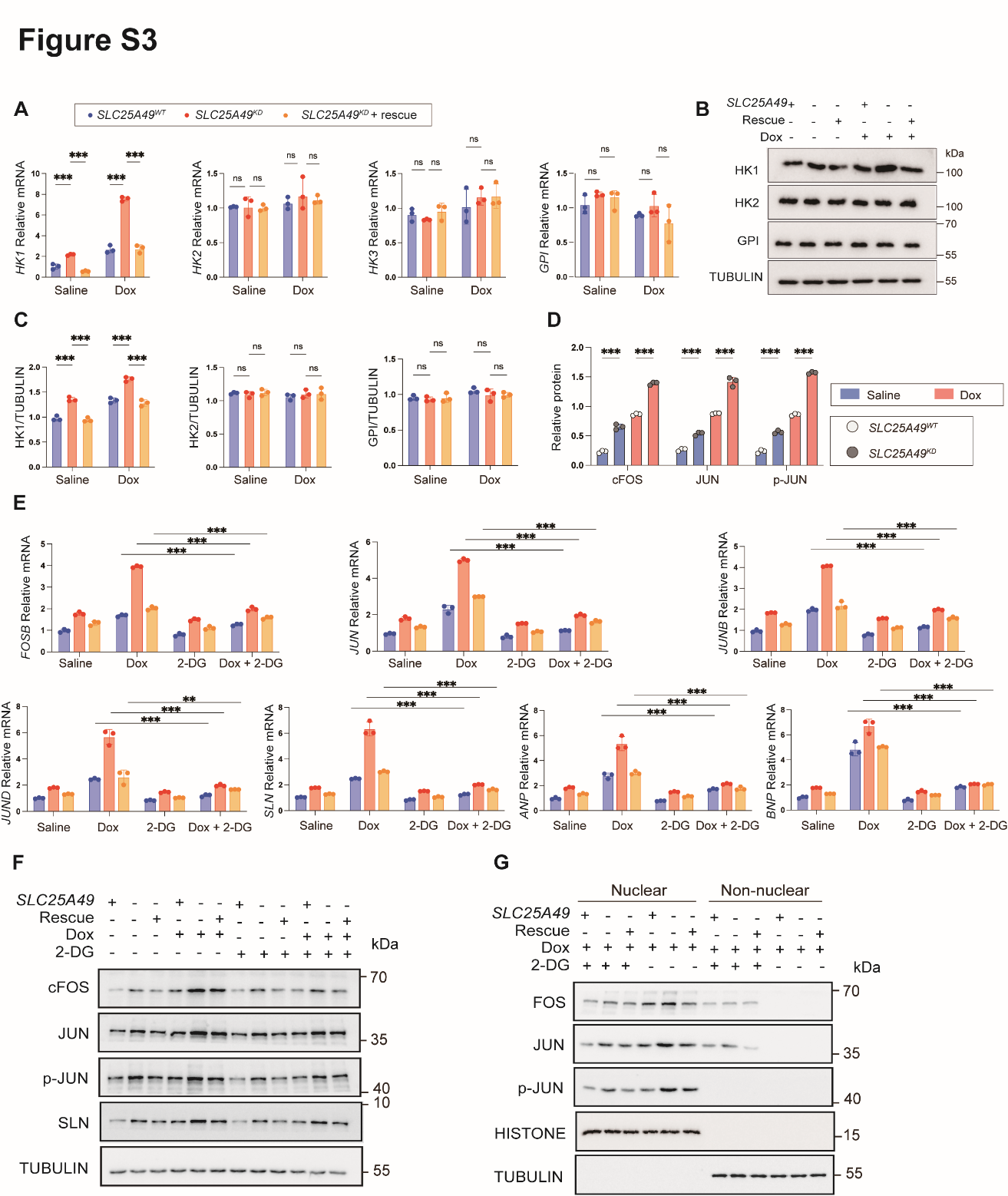
**

**Figure S3. *SLC25A49^KD^* upregulates glycolysis and AP-1 expression in human cardiomyocytes.** (A) Real-time quantitative PCR analysis determining the mRNA expression of G6P-related genes during glycolysis in AC16 clones of *SLC25A49^WT^*, *SLC25A49^KD^*, and *SLC25A49^KD^* + rescue treated with or without Dox for 24 hours (Dox, 500 nM; n = 3 for each group). (B) Representative immunoblotting images showing G6P-related protein during glycolysis in AC16 clones of *SLC25A49^WT^*, *SLC25A49^KD^*, and *SLC25A49^KD^* + rescue treated with or without Dox for 24 hours (Dox, 500 nM; n = 3 for each group). (C) Quantification of (B). (D) Quantification of Figure 4F. (E) Real-time quantitative PCR determining the mRNA expression of AP-1-related genes and *SLN* gene in AC16 clones of *SLC25A49^WT^*, *SLC25A49^KD^*, and *SLC25A49^KD^* + rescue treated with Dox, 2-DG, 2-DG puls Dox, or control for 24 hours (Dox, 500 nM; 2-DG, 50 mM; n = 3 for each group). (F-G) AC16 clones of *SLC25A49^WT^*, *SLC25A49^KD^*, and *SLC25A49^KD^* + rescue were treated with Dox, 2-DG, 2-DG puls Dox, or control for 24 hours. AP-1 family proteins and SLN protein were measured by immunoblotting (F), and nucleocytoplasmic separation (G) (Dox, 500 nM; 2-DG, 50 mM; n = 3 for each group). G6P, glucose-6-phosphate; SLN, sarcolipin, 2-DG, 2-Deoxy-D-glucose. Data were presented as means ± SD, and analyzed by one-way ANOVA test. ns, not significant, ***P* < 0.01, and ****P* < 0.001.


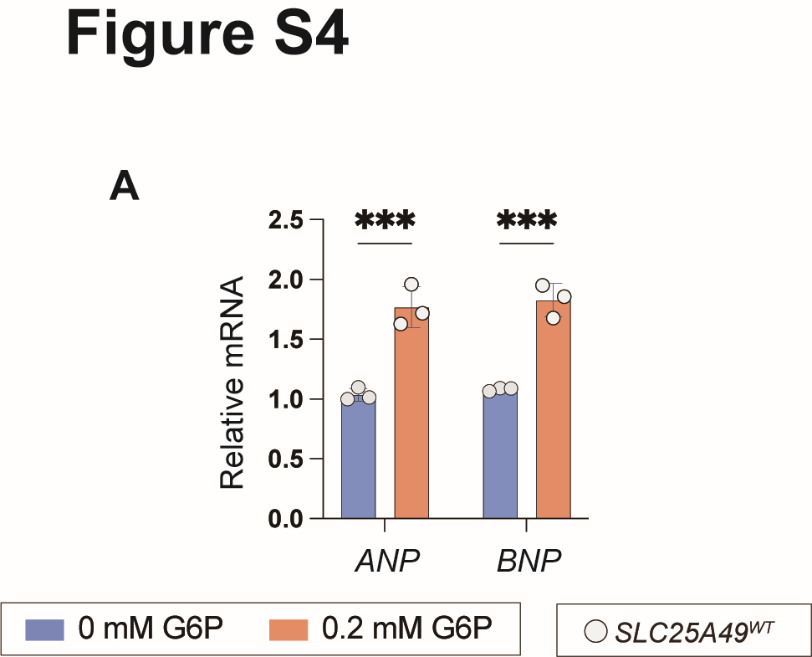


**Figure S4. G6P contributes to cardiac injury in human cardiomyocytes.** (A) Real-time quantitative PCR analysis determining the mRNA expression of *ANP* and *BNP* genes in AC16 clones treated with 0.2 mM G6P or control for 24 hours (n = 3 for each group). G6P, glucose-6-phosphate. Data were presented as means ± SD, and analyzed by t test. ****P* < 0.001.


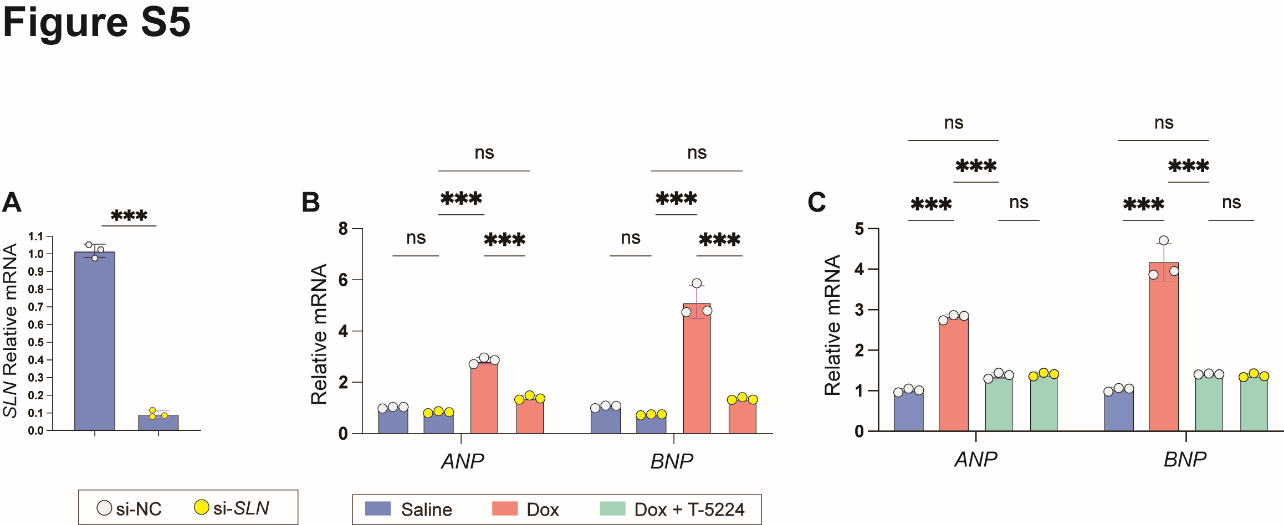


**Figure S5. SLN aggravates myocardial injury in human cardiomyocytes.** (A) Real-time quantitative PCR analysis determining the mRNA expression of *SLN* in AC16 clones treated with si-*SLN* or a scramble control (n = 3 for each group). (B) Real-time quantitative PCR analysis determining the mRNA expression of *ANP* and *BNP* in AC16 clones treated with *SLN* siRNA under saline or Dox induction for 24 hours (n = 3 for each group). (C) Real-time quantitative PCR analysis determining the mRNA expression of *ANP* and *BNP* in AC16 clones treated with *SLN* siRNA under saline, Dox, or Dox plus T-5224 induction for 24 hours (n = 3 for each group). Data were presented as means ± SD, and analyzed by t test and one-way ANOVA test. ns, not significant, and ****P* < 0.001.


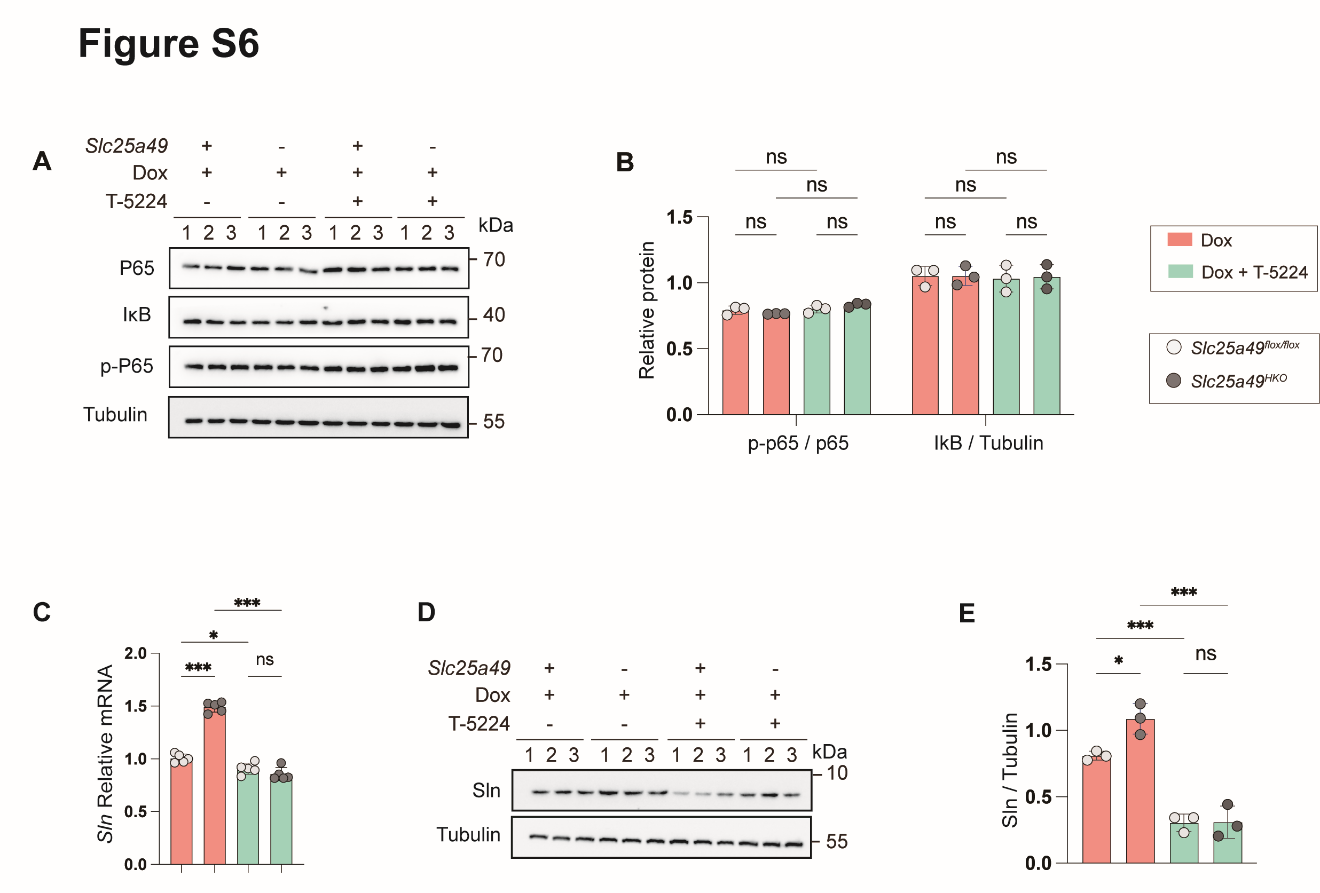


**Figure S6. T-5224 inhibits Dox-induced Sln expression, without effect on NF-κB signaling pathway.** (A) Representative immunoblotting images showing NF-κB related protein in Dox-treated *Slc25a49^flox/flox^* and *Slc25a49^HKO^* hearts with or without T-5224 induction at 3 months of age (n = 3 for each group). (B) Quantification of (A). (C) Real-time quantitative PCR analysis determining the mRNA expression of *Sln* genes in four groups of mice (n = 3 for each group). (D) Representative immunoblotting images showing Sln protein in four groups of mice (n = 3 for each group). (E) Quantification of (D). Data were presented as means ± SD, and analyzed by one-way ANOVA test. ns, not significant, **P* < 0.05, and ****P* < 0.001.

**Table S1. Sequences of siRNA/shRNA used in this study.**

| **Gene symbol** | **Sequence (5' to 3')** |
| --- | --- |
| Human-shRNA-*NC* | GATCCGTTCTCCGAACGTGTCACGTAATTCAAGAGATTACGTGACACGTTCGGAGAATTTTTTC |
| Human-shRNA-*SLC25A49* | GATCCGCCGGGTGTCATCAGGATCATCTCGAGATGATCCTGATGACACCCGGCTTTTTTG |
| Human-siRNA-*NC* | UUCUCCGAACGUGUCACGUdTdT |
| Human-siRNA-*SLN* | GCAACCAAACUCUAAUUCAdTdT |

**Table S2. Primers used for the PCR assays in this study.**

| **Primer** | **Sequence (5' to 3')** |
| --- | --- |
| Mouse-*Anp*-forward | GCTTCCAGGCCATATTGGAG |
| Mouse-*Anp*-reverse | GGGGGCATGACCTCATCTT |
| Mouse-*Bnp*-forward | GAGGTCACTCCTATCCTCTGG |
| Mouse-*Bnp*-reverse | GCCATTTCCTCCGACTTTTCTC |
| Mouse-*Col1a1*-forward | GCTCCTCTTAGGGGCCACT |
| Mouse-*Col1a1*-reverse | ATTGGGGACCCTTAGGCCAT |
| Mouse-*Col5a1-forward* | CTTCGCCGCTACTCCTGTTC |
| Mouse-*Col5a1-reverse* | CCCTGAGGGCAAATTGTGAAAA |
| Mouse-*Mmp2-forward* | ACCTGAACACTTTCTATGGCTG |
| Mouse-*Mmp2-reverse* | CTTCCGCATGGTCTCGATG |
| Mouse-*Ndufs8-forward* | GTGGCGGCAACGTACAAGTAT |
| Mouse-*Ndufs8-reverse* | GAATCCGAGCTGCATTGTCAG |
| Mouse-*Ndufv3-forward* | GAGAGGGGCAAGCTCCTAAC |
| Mouse-*Ndufv3-reverse* | ACGCTACCAAAGTCTTTCTTGAC |
| Mouse-*Sdhb-forward* | ATTTACCGATGGGACCCAGAC |
| Mouse-*Sdhb-reverse* | GTCCGCACTTATTCAGATCCAC |
| Mouse-*Sdhc-forward* | GCTGCGTTCTTGCTGAGACA |
| Mouse-*Sdhc-reverse* | ATCTCCTCCTTAGCTGTGGTT |
| Mouse-*Uqcc1-forward* | TTGCTGGTGCGAGTCCTTAG |
| Mouse-*Uqcc1-reverse* | GTGTCCGCTCCAACAGTCT |
| Mouse-*Uqcc2-forward* | TCCGGGAGGGAGAGAACAC |
| Mouse-*Uqcc2-reverse* | AGGGTACTTGTGCTTGTAGTAGT |
| Mouse-*Cox6a1-forward* | TCAACGTGTTCCTCAAGTCGC |
| Mouse-*Cox6a1-reverse* | AGGGTATGGTTACCGTCTCCC |
| Mouse-*Cox7a1-forward* | GCTCTGGTCCGGTCTTTTAGC |
| Mouse-*Cox7a1-reverse* | GTACTGGGAGGTCATTGTCGG |
| Mouse-*Atp1b2-forward* | CAGACCGTCTCTGACCATACC |
| Mouse-*Atp1b2-reverse* | TCTCAGTCTTGGGTCGAATCA |
| Mouse-*Atp8b2-forward* | GGGCTAACGACCGTGAATACA |
| Mouse-*Atp8b2-reverse* | GAGGACGAGAACCAAAGGCA |
| Mouse-*Atpaf1-forward* | CCCCTTCTACGACCGCTAC |
| Mouse-*Atpaf1-reverse* | CCACTGGCTGCTTTCGGAA |
| Mouse-*Hk1-forward* | AACGGCCTCCGTCAAGATG |
| Mouse-*Hk1-reverse* | GCCGAGATCCAGTGCAATG |
| Mouse-*Hk2-forward* | ATGATCGCCTGCTTATTCACG |
| Mouse-*Hk2-reverse* | CGCCTAGAAATCTCCAGAAGGG |
| Mouse-*Hk3-forward* | TGCTGCCCACATACGTGAG |
| Mouse-*Hk3-reverse* | GCCTGTCAGTGTTACCCACAA |
| Mouse-*Gpi-forward* | CTCAAGCTGCGCGAACTTTTT |
| Mouse-*Gpi-reverse* | GGTTCTTGGAGTAGTCCACCAG |
| Mouse-*Fos-forward* | CCCGAGCTGGTGCATTACA |
| Mouse-*Fos-reverse* | GAGGCCAGATGTGGATGCTT |
| Mouse-*Fosb-forward* | CCTTCAGTCCCAAAGACGAGT |
| Mouse-*Fosb-reverse* | GGGTGGGGTTTGGGATTAGG |
| Mouse-*Jun-forward* | CAGACTGTACACCAGAAGATGGT |
| Mouse-*Jun-reverse* | AAACTTCCATGGGTCCCTGC |
| Mouse-*Junb-forward* | TATAAAAGCTTGGGGCTGGGG |
| Mouse-*Junb-reverse* | GGGCTTCCGGAGATTTTCTCT |
| Mouse-*Jund-forward* | GAAACGCCCTTCTATGGCGA |
| Mouse-*Jund-reverse* | CAGCGCGTCTTTCTTCAGC |
| Mouse-*Sln-forward* | TCAGGAAGTGAAGACAAGCC |
| Mouse-*Sln-reverse* | GGAGCCACATAAGGAGAACG |
| Mouse-*Actb-forward* | GTGACGTTGACATCCGTAAAGA |
| Mouse-*Actb-reverse* | GCCGGACTCATCGTACTCC |
| Human-*FOS-forward* | CCGGGGATAGCCTCTCTTACT |
| Human-*FOS-reverse* | CCAGGTCCGTGCAGAAGTC |
| Human-*FOSB-forward* | GCTGCAAGATCCCCTACGAAG |
| Human-*FOSB-reverse* | ACGAAGAAGTGTACGAAGGGTT |
| Human-*JUN-forward* | TCCAAGTGCCGAAAAAGGAAG |
| Human-*JUN-reverse* | CGAGTTCTGAGCTTTCAAGGT |
| Human-*JUNB-forward* | ACGACTCATACACAGCTACGG |
| Human-*JUNB-reverse* | GCTCGGTTTCAGGAGTTTGTAGT |
| Human-*JUND-forward* | TCATCATCCAGTCCAACGGG |
| Human-*JUND-reverse* | TTCTGCTTGTGTAAATCCTCCAG |
| Human-*SLN-forward* | ATGGTCCTGGGATTGACTGAG |
| Human-*SLN-reverse* | GTGCCCTCGGATGGAGAATG |
| Human-*ACTB-forward* | CTCACCATGGATGATGATATCGC |
| Human-*ACTB-reverse* | AGGAATCCTTCTGACCCATGCC |
| Human-*ANP-forward* | CAACGCAGACCTGATGGATTT |
| Human-*ANP-reverse* | AGCCCCCGCTTCTTCATTC |
| Human-*BNP-forward* | TGGAAACGTCCGGGTTACAG |
| Human*-BNP-reverse* | CTGATCCGGTCCATCTTCCT |
| Human-*SLN-forward* | CAAGCCGCTGTGAAAATGG |
| Human*-SLN-reverse* | GAGCATCTCAGTCAATCCCAG |
| Human-CHIP2300-forward | ACAGCTAACCAGGCACAACA |
| Human-CHIP2300-reverse | TGCCTGACAGTAGTAGAACGTG |
| Human-CHIP1100-forward | CGTCCTTGTCTGATTTGAGCC |
| Human-CHIP1100-reverse | TACAGAAGCCTGGGAGGTTG |
